# Subway tunnels displacement analysis due to two different communication channels construction procedures

**DOI:** 10.1016/j.heliyon.2019.e01949

**Published:** 2019-06-17

**Authors:** Massamba Fall, Zhengguo Gao, Becaye Cissokho Ndiaye

**Affiliations:** Dept. of Transportation and Civil Engineering, Beihang University, Beijing 100083, China

**Keywords:** Structural engineering, Subway station, Construction sequence, Communications channel, Numerical simulation, Displacement

## Abstract

Due to congestion in overground space, many underground structures have been built to facilitate communications between cities. These structures constructions will induce soil displacement, cities planning rearrangement, and sometimes are near to future buildings constructions. In some cases, communication channels should be built to allow the underground junction between the building and the subway station. Numerical simulation has been carried out to study the tunnel displacement behavior during construction stages. Two different procedures of the communication channel construction were also investigated. The results reliability were evaluated by monitoring six points displacement in the sheet pile walls after completion of the foundation pit. The results obtained were reasonably close to the monitoring results proven reasonably of the research ideas and the method scientificity. The results showed using sheet piles walls, and struts before soil excavation could reduce considerably the lateral displacement of the subway line during the excavation process and induce augmentation in the subway line vertical displacement. The lateral displacement effect of the walls thickness variation is much superior to the one provides by the struts cross-section and pile's diameter variations.

## 1 Introduction

Frequently utilized in construction insurance or human safety, deformation monitoring inspections are one of the most significant engineering surveying activities [1]. Their investigations can increase our knowledge of the structures characteristics deformation and can aid improvements to engineering designs. They are required for the secure operation of engineered structures such as viaducts, earthworks, dams, light rail transit systems, tunnel safety analysis, bridges, and high-rise building [2]. Nowadays many tunnels (Underground structures) have been built due to congestion in over ground space to facilitate communications between cities. Residential and commercial zones will certainly develop after the tunnel subways line construction. When the buildings are near to the subway line, communications channels could be constructed as a junction between the subway and the buildings. Different constructions procedures could be adopted for the construction of the communication structures. Many excavations have been done near subways line. Those excavations affect the soil and surrounding structures nearby, especially in high water, soft soil, and in alternative conditions. In these cases, foundation pit excavations cause calamities, such as foundation failure, soil slip, a severe leakage of retaining structures, deflection of the pile, foundation uplift, stream soil, and others, etc., which may be a sincere menace to the safety of the adjacent surrounding structures, over and underground buildings.

Numerical simulation can be an impressive tool to simulate the excavation and construction sequence induce ground movement [3, 4]. Before starting the actual calculations, the initial conditions must be generated. Generally, the initial stress state and the initial geometry configuration comprise the initial conditions. Depending on the model configuration, many assumptions could be drawn to evaluate the initial stress state. When subway tunnels are in operation, the surrounding soil is stable. Once the excavation is executed nearby, the upper periphery will be unloaded, the soil around the tunnel will become loose with a decreasing bearing capacity [5].

Many researchers studied excavation and construction sequence induce subways tunnels and surrounding soil displacement [6, 7, 8, 9] when a tunnel is located directly beneath an excavation; it will move upward. However, additional measured such as monitoring results can be used to ensure the reliability of the numerical simulation and the conclusions drew from the simulation. Jin [10] studied a case of two existing subway tunnels under crossed by four closely spaced tunnel in Shenzhen. The calculation results match well with the measured tunnel settlement data and accuracy of the proposed numerical method is verified. The horizontal displacement is less affected by the new tunnels underneath construction compared with the existing tunnels vertical displacement. The excavation completion should be done within a limited duration, followed by the struts immediately and properly propping and over-excavation should avoid, particularly when the depth is higher. Otherwise, over-excavation, long construction duration time and, a long duration time of unpropped wall exposure will induce important ground and retaining wall movements Tan [11]. This justifies the necessity of studying the safety of existing tunnel during new structures construction. Tests showed that displacement measurement by the traditional methods is less convenient than by a total station. Measurement of the crown settlement by a total station is with 3D coordinates measurements produces an error, which accounts for a 13% difference in the total crown settlement Luo [12]. Zhang [13] studied the health monitoring on the deformation of operating metro shield tunnels influenced by a close deep excavation. Both manual and automatic method was adopted in the safety monitoring during deep excavation. The comparison shows that automatic monitoring results could be affected by poor working conditions and cumulative error. In light of this, the numerical simulation and field monitoring results are not expected to be the same values in the construction sequence case. Unfortunately, both calculations procedures involve errors due to their applications. However, the numerical simulation results obtained should be reasonably close to the monitoring results to ensure the reliability of the calculations [4, 14, 15, 16, 17].

Tunnel deformation due to new structures construction is a typical problem of soil structure interaction. Under lateral loading, Abedzadeh [18] presented a rigorous mathematical treatment of an embedded pile within the three-dimensional elastostatics and the engineering beam theory. The author derived new analytical insights and benchmark numerical results for the soil–structure interaction problem. Kitiyodom [19] used Mindlin's solutions to study the soil-pile interaction during piled raft foundations deformation subjected to ground movement. The reliability of the solution was verified through some published solutions.

In this paper, three-dimensional numerical simulations of foundation pit repair, connection channel excavation, construction procedures, and building construction sequence are presented. All these procedures involve the pile's construction, retaining walls construction, anchors installation, and construction sequence definition. The communications structures linked the subway line and the building structure. Two different procedures have been evaluated for its construction. Since 2015, the foundation pit was completed, and the displacement of the walls was evaluated in some points by total station. Before the actual construction, those results were compared with the numerical calculations values. The numerical simulation results were close enough to the filed monitoring data to ensure the reliability of the results and the conclusions obtained after project completion.

## 2 Background

### 2.1 Project overview

**Fig. 1:**
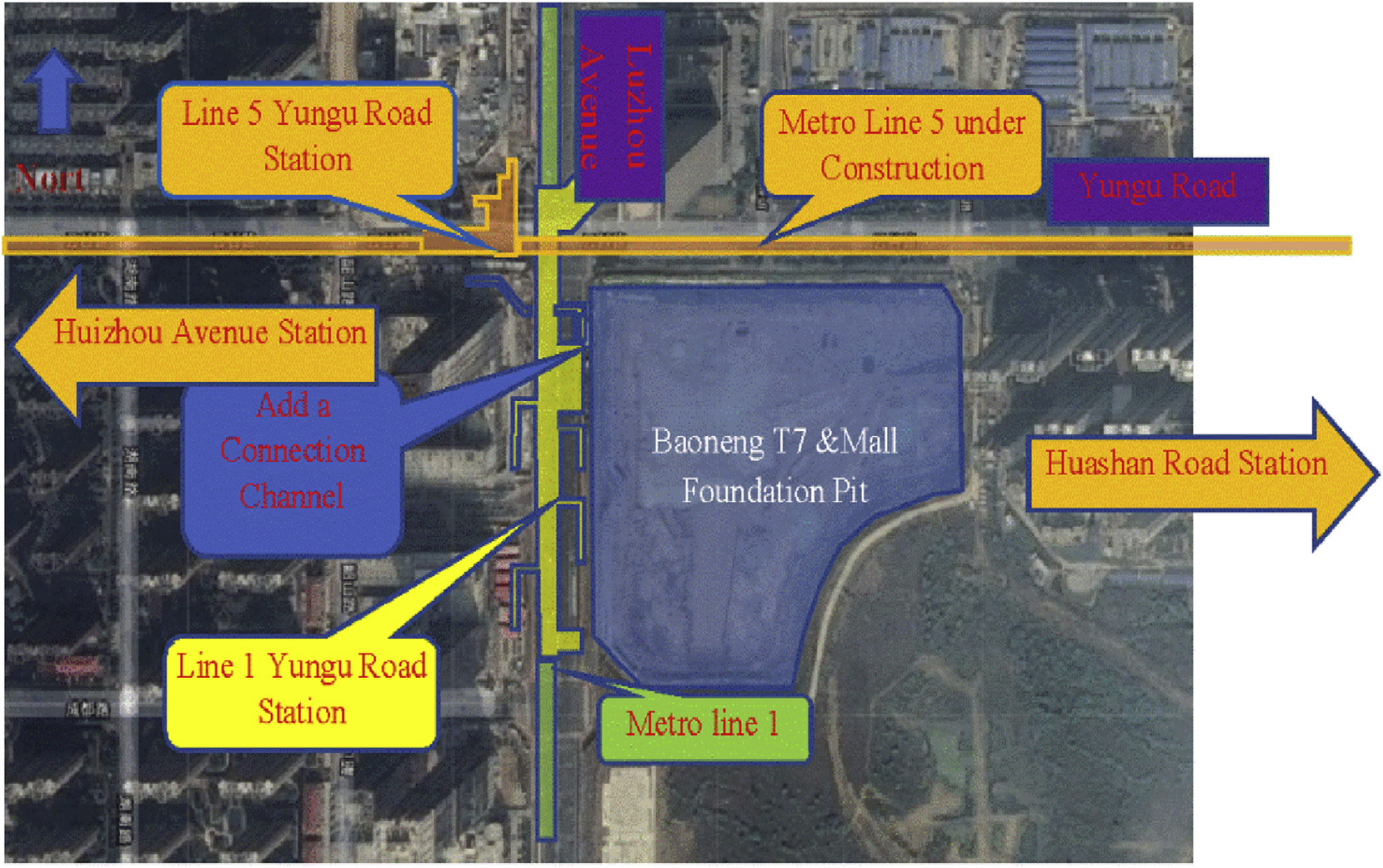
Baoneng T7&MALL project and rail transit location map.

The location of Baoneng T7&MALL and Metro Lines 1 and 5 is shown in Fig. 1. The Baoneng T7&MALL foundation pit reinforcement and the new communication channel are within the scope of the subway station control and protection zone, and its construction may affect the safety of the rail transit structure under operation and construction. It is necessary to carry out the rail transit safety caused by the implementation of this project. Impact assessment. It is predicted that the excavation of the foundation pit and the communication channel will cause the deformation of the rail transit structure, and based on the evaluation results, the recommended values and protection measures for the monitoring indicators of the rail transit structure during the construction period are proposed.

(1) Establish a three-dimensional finite element model, simulate the foundation pit reinforcement and communication channel construction process of Baoneng T7&MALL project, and provide analysis results of the main body and subsidiary structure of the Yungu Road Station of Metro Line 1 and the deformation of the tunnel of Metro Line 5 To assess the impact on its safety.
(2) Give various control indicators for station structure and track structure deformation of subway lines 1 and 5 during the construction of the pit reinforcement and communication channel of Baoneng T7&MALL project.

**Fig. 2:**
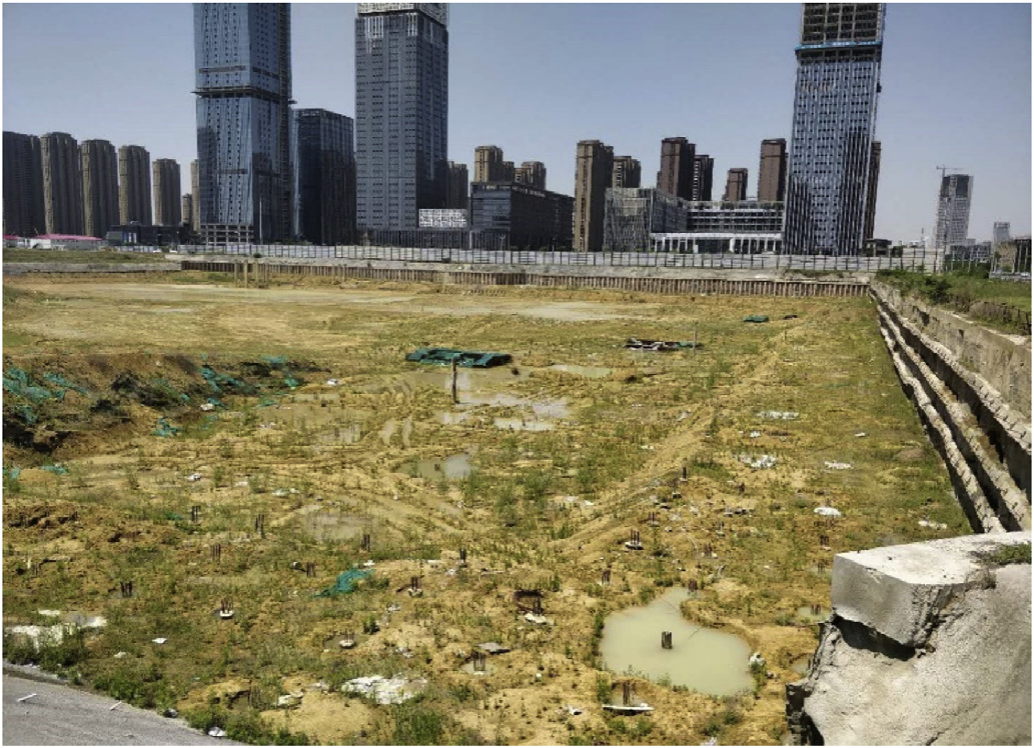
Status of foundation pit.

The foundation pit of Baoneng T7&MALL project is located on the southeast side of the intersection of Yungu Road and Yinzhou Avenue in Binhu New District of Hefei City. The foundation pit protection was completed in 2015 (status od foundation pit now Fig. 2). It has passed the design and has been used for field investigation. The foundation pit retaining structure is damaged to varying degrees, and the foundation pit must be re-reinforced. A new underground connecting section is connected to the entrance and exit of No. 2 Yungu Road Station of Metro Line 1.

Yungu Road Station of Metro Line 1 is an operator station with a north-south direction of 468.5m and an east-west direction of about 21m. The vehicle is a two-story, three-span, two-column station, which is connected to the station of Line 1 at the station floor through a communication channel.

Metro Line 5 is the line under construction. At present, the construction of Yungu Road Station on Line 5 has been completed. The station is a three-story, three-span and, two-story underground station. The Huashan Road Station and Yungu Wheel Section are constructed by shield method. Directions from Huashan Road Station to Yungu Road Station, the left line (north side) has been received, and the right line (south side) is about 500m away from Yungu Road Station.

### 2.2 Subway and walls material properties

According to the data of the foundation engineering design, the foundation pit crown beam and the support pile are made of C30 concrete, and the steel bars are HPB300, HRB335, and HRB400. The basic mechanical parameters of the material for numerical calculation could be observed in (Table 1).

**Table 1:** Structural materials properties.

	Young's Modulus (kPa)	Poisson' s ratio
Metro station C30 reinforced concrete beam and column:	3.00E+07	0.25
Subway station C30 reinforced concrete wall structure:	3.00E+07	0.25
Foundation pit concrete cushion:	2.60E+07	0.27
Foundation pit concrete support pile:	3.00E+07	0.25
Foundation pit anchor steel cable:	2.10E+08	0.2

The behavior of all the subway walls is assumed to be linear during the simulation stages. The walls were modeled with 4-node doubly curved thin or thick shell, reduced integration, hourglass control, finite membrane strains (S4R5), and the material is concrete (Table 1). All the beam, columns, piles, and supports elements in the model are modeled with a 2-node linear beam in space (B31) available in Abaqus.

### 2.3 Soil properties

The establishment of the site finite element model is based on the engineering geological data and topographical conditions. The computed results by FEM are dependent on constitutive soil models. In the present study, the Mohr-Columb model (MC model) was adopted to capture the behavior of the soil layers. This model was used by previous authors to study the effect of excavation on an existing metro tunnel in a complicated environment [4, 20]. The results were acceptable to allow the use of this model in this kind of problem. The frictional angle and cohesion of the soil material are selected according to the mechanical recommended parameter (because the engineering recommended parameters have been safe). The site is composed of six types of soil layers Table 2. The soil parameters were obtained by carrying out laboratory tests. 8-node linear brick elements (C3D8R) with reduced integration is used to model the soil layers, and hourglass control coefficients reduction are directly selected in the model calculation. The geological stratification of the construction site is shown below in Table 2.

**Table 2:** Selection of physical and mechanical parameters of site soil.

Layers	Rock and Soil Mass	Density (kN/m3)	Pore ratio	Compression modulus (MPa)	Cohesion (kPa)	Internal friction angle (°)	Permeability (cm/s)	Elastic modulus (kPa)	Poisson's ratio
1	Prime fill	18.86	0.858	3.4	8	12	5.00E-05	17000	0.3
2	Clay 1	19.37	0.792	5.1	21	30	2.00E-06	25500	0.26
3	Clay 2	19.37	0.72	6.4	21	30	5.00E-05	32000	0.28
4	Silty Clay	20.18	0.599	7.8	15	33	5.00E-05	39000	0.28
5	Stringly Weathered sandy Mudstone	20.49	0.6	20	14.5	55	1.00E-05	100000	0.26
6	Middle Weathered Sandy Mudstone	20.49	0.6	20	14.5	55	1.00E-05	100000	0.26

According to the design requirements, the site foundation pit was completed two years ago, and there is no significant change in groundwater level during the construction phase, ignoring the impact of groundwater level changes on the site structure deformation. Fig. 4 shows the geological model of the construction site. Fig. 5 shows the whole model after achievement of the above-ground structure.

**Fig. 3:**
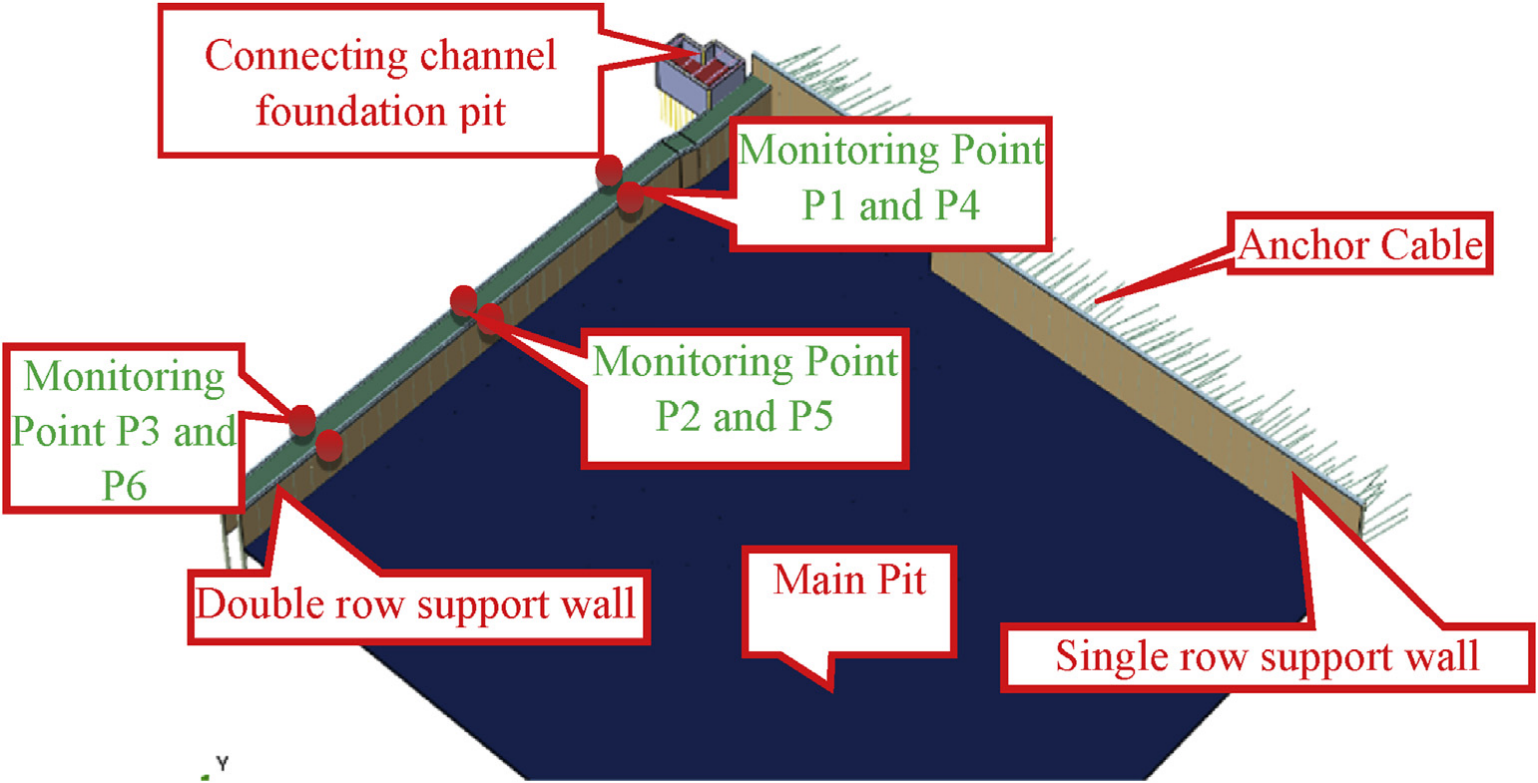
Finite element model of foundation pit structure.

**Fig. 4:**
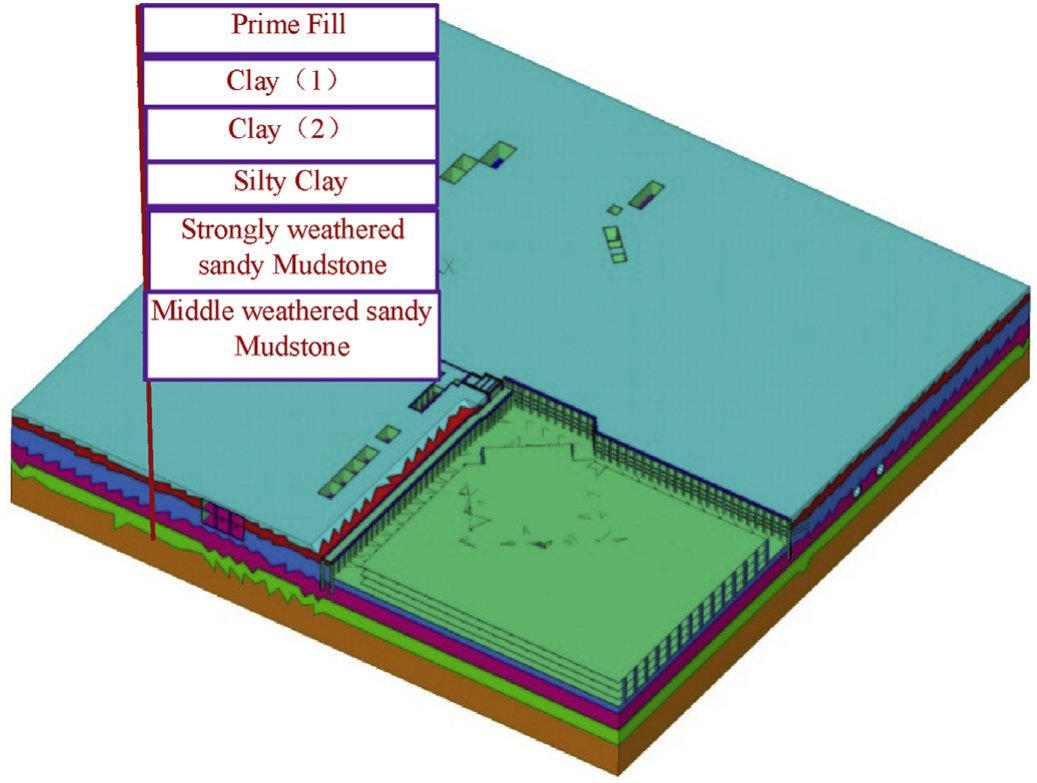
Geological model of the construction site.

**Fig. 5:**
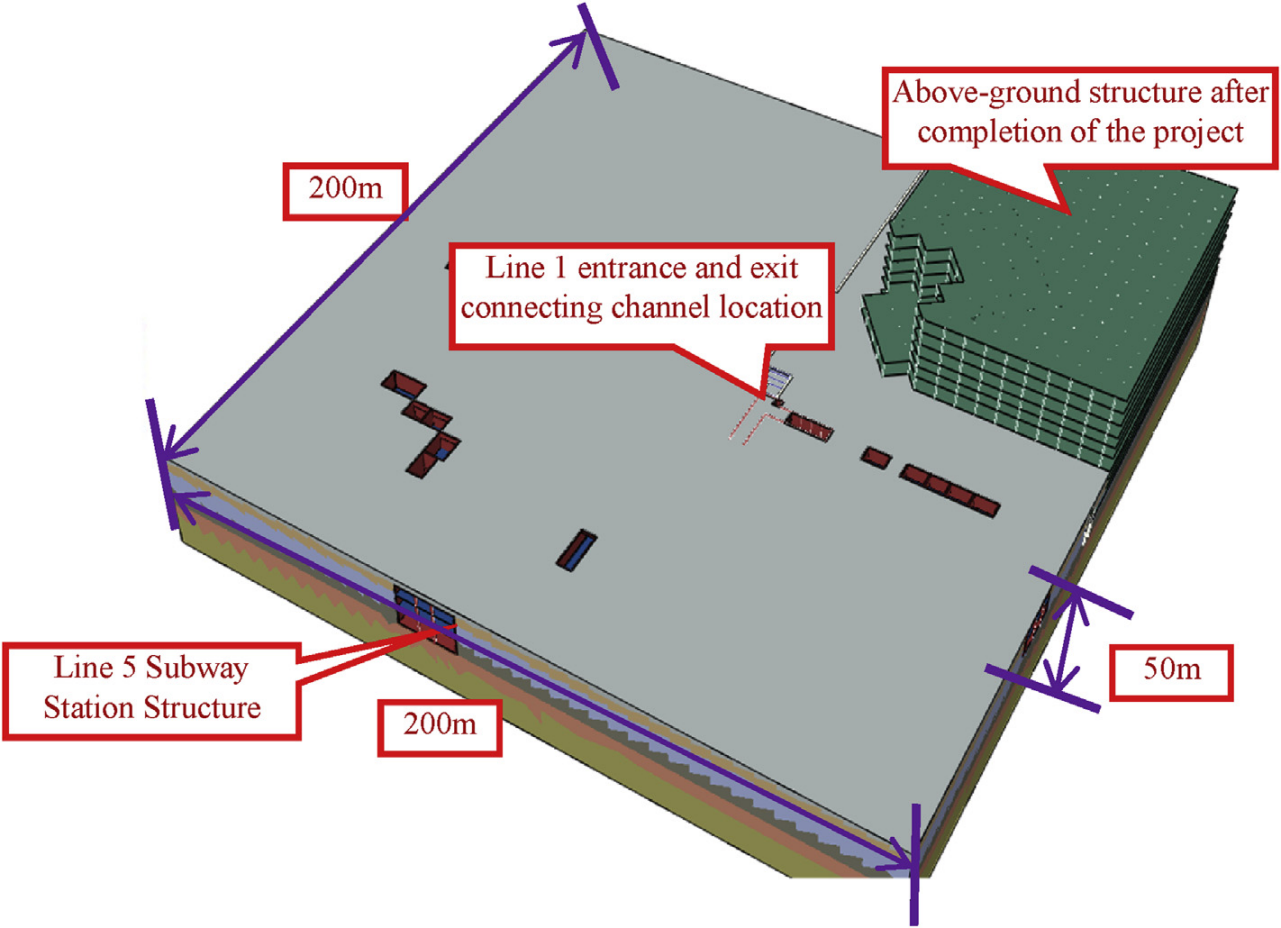
Finite element model of the overall structure of the site.

### 2.4 Field monitoring results evaluation

Methods for estimating excavation-induced ground movements are mainly separated into three categories: empirical methods, analytical solutions, and numerical methods. In the empirical methods, the greenfield settlement profile is usually approximated by an inversed Gaussian curve [21, 22]. They cannot provide stresses and deformations for the entire model. The Analytical solutions can be a simple tool for reasonable predicting ground settlement profile and can offer a rapid analysis of the stress and strain fields and can fit with the values of the measures [23]. However, it seems not suitable and complicates for detailed analysis in some cases like plasticity, creep, nonlinear soil behavior [24]. In some cases, the results getting from the analytical method are nearly consistent with the results from numerical methods [3].

Numerical simulation can be used to study the effects of excavation and construction sequence on existing structures. However, the results got from the numerical simulation must be compared to some monitoring values to ensure their reliability. In the light of this, Six points have been chosen at the top of the sheet piles walls (Fig. 3) to evaluate their horizontal displacement using a total station (an electronic/optical apparatus used in current surveying and building construction that uses electronic distance meter (EDM) in conjunction with electronic transit theodolite. It is also integrated with electronic data collector, storage system, and microprocessor).

Total station is used to evaluate a sloping distance of an object to the apparatus, vertical and horizontal angles. This Microprocessor unit permits for data collected calculation to obtain the horizontal distance, a point coordinates and reduced the point level [25]. Data collected from the total station can be downloaded into computer/laptops for further processing of information. Civil engineers and land surveyors mainly use either total stations, either to set out features (such as houses, roads, or boundaries) or record features as in topographic surveying.

### 2.5 Assumptions

(1) Metro Line 1 station only considers normal working conditions. Due to the relatively short construction period, accidental loads such as civil air defense and earthquakes are not considered.
(2) The right line of the tunnel of Yungu Road Station to Huashan Road Station of Metro Line 5 has been completed, and the left line is still under construction. Considering that the implementation period of the project is relatively lagging, from the conservative considerations, the model considers that the left and right lines are all connected.
(3) It is assumed that the concrete structure such as the interval tunnel is an elastic material, the soil is an elastic plastic material and a Mohr-Coulomb model is adopted.
(4) It is assumed that the interval tunnel structure and the soil conform to the principle of deformation coordination.
(5) The premise of this evaluation analysis is that the construction is under normal and good control conditions.

## 3 Calculation

### 3.1 Method

This evaluation principally uses numerical simulation to determine and analyze stress and displacement predictions. This evaluation supposes that the Metro Line 1 station is already in a stable state. The analysis results are just the supplementary deformations due to the execution of the project on subway lines 1 and 5.

Two types of model are generally used in the literature for the calculation:

(1) soil-structure (Continuum) model; and
(2) load-structure (action-reaction) model.

The soil-structure model is generally used for deformation evaluation due to the stratum-structure interaction. For deformation analysis and structural internal force evaluation, the action-reaction model can be used. The structure is just considered as a calculation item.

The stratum-structure model is carried out because the model is firmly about the stratum given because of the subway line settlement that occurred during the construction stages.

ABAQUS/CAE software is used for deformation evaluation. It is universally considered as an effective tool for finite element analysis. It can analyze complicated structural and solid mechanics, principally to handle very complex and large cases and simulate deeply nonlinear problems. Abaqus can be carried out in the accurate modeling and analysis of several practical issues like foundation pits, hydraulics, tunnels, subways, slopes, pile foundations, and mines. Many authors used this software successfully to evaluate soil displacement due to excavation [16,26,27]. The results were reasonably close to the monitoring values.

The simulation stages are defined through the birth and death process. All domains must be set initially, and those zones are corresponding to future construction should be deleted in the initial phase. In this case, all stresses, pore pressures, etc…. in the affected areas are set to zero. During the simulation, the newly constructed areas are added in the model following the defined sequence.

### 3.2 Calculations conditions and cases study

**Fig. 6:**
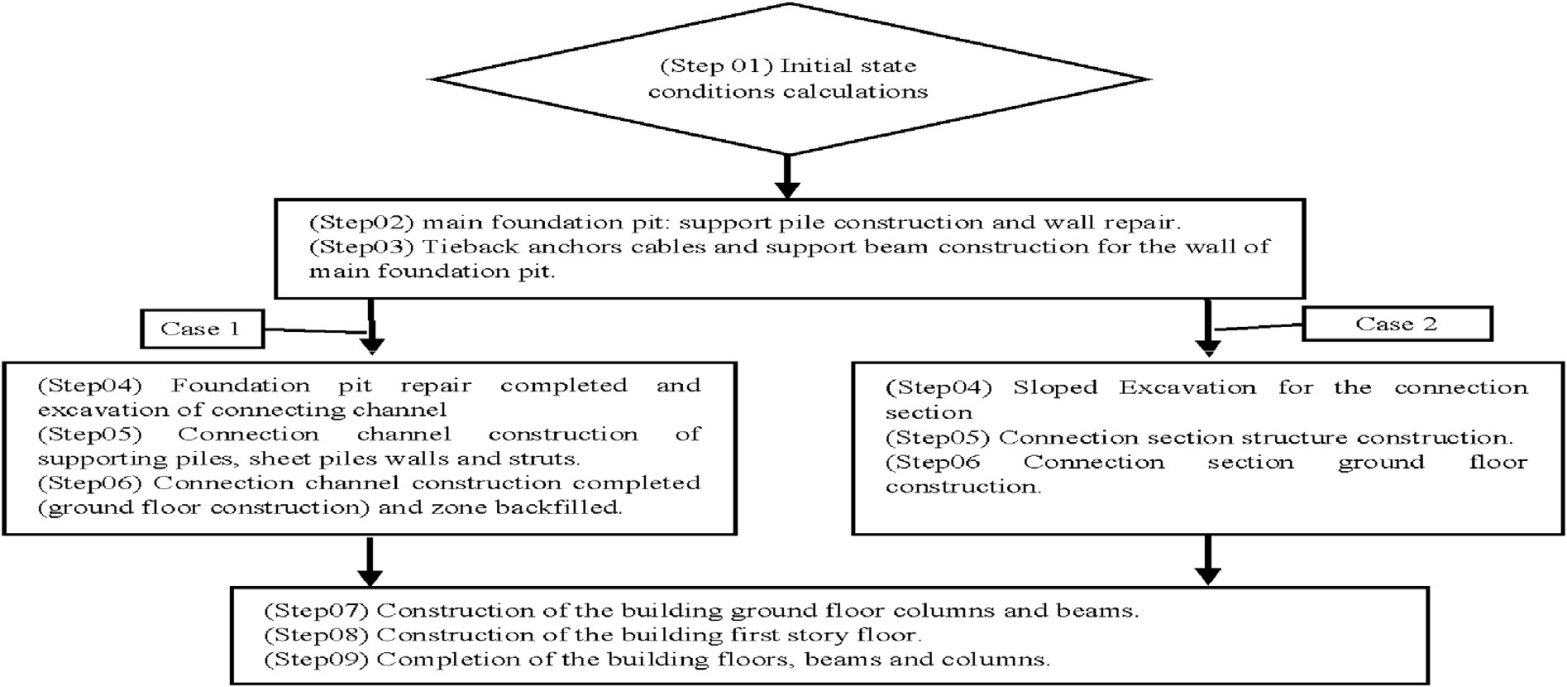
Construction procedure.

This paper simulated the foundation pit reinforcement, a connecting channel foundation pit excavation, a communication channel construction, backfilling, basement structure construction and other operations (Fig. 6) on the structure of Metro Line 1 and Line 5.

**Fig. 7:**
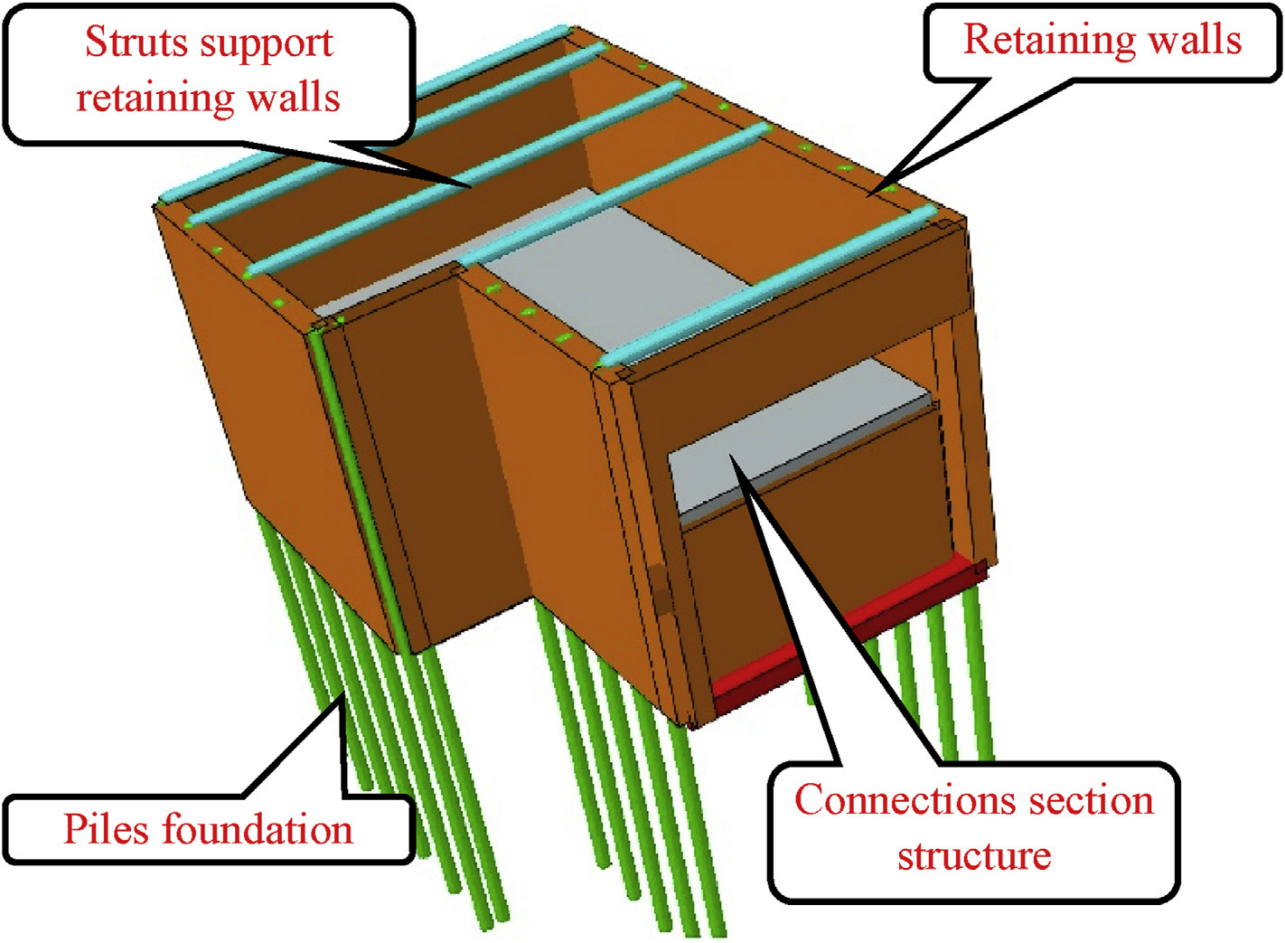
Case 1. Connection section construction after braced excavation vremove: 732.62m3.

In case 1, before construction of the connection channel, retaining walls combine with struts have been used to support the soil after excavation. Concrete piles had also been used to increase the stability of the walls and to support the connection channel structure (Fig. 7).

**Fig. 8:**
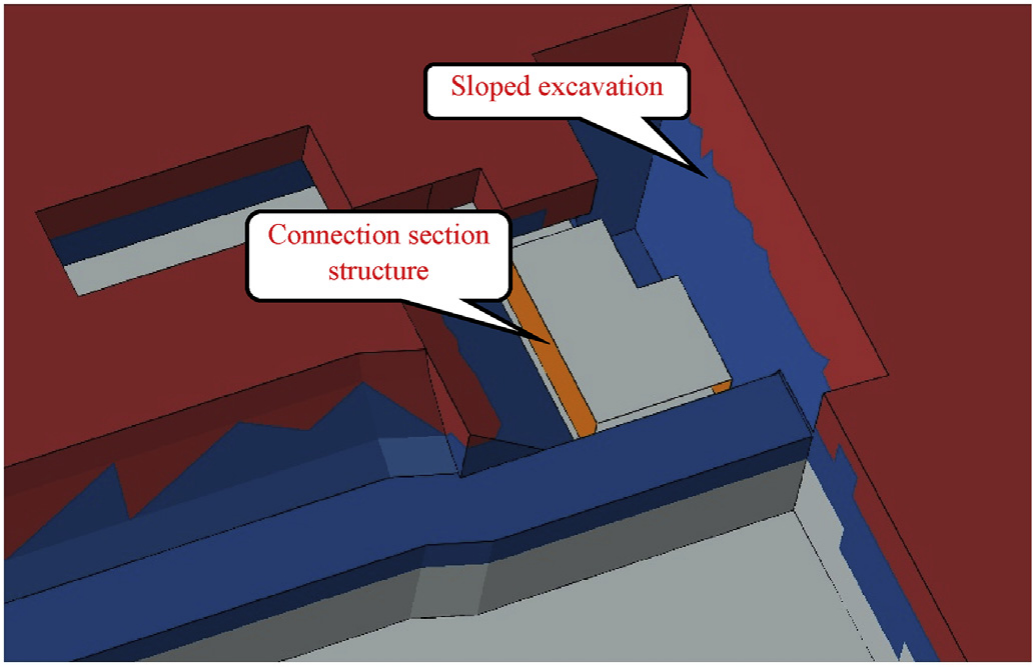
Case 2. Connection section construction vremove: 1830.84m3.

However, in the case 2, sloped excavation was used to build the connection section. Although sloping during the excavation process can avoid the soil from pouring due gravity load resistance effect (Fig. 8).

The case 1 procedure has been adopted in real engineering and the results have been compared to the field monitoring values obtained by total station measured.

## 4 Results and discussion

### 4.1 Initial state simulation

In all civil or mining engineering projects, there is an in-situ state of stress in the ground, before any excavation or construction is started. This is the state of all variables in the model (e.g., stresses or pore pressures) before any loading change or disturbance. Ideally, information about the initial state comes from field measurements. But, when these are not available, the model can be run for a range of possible conditions. Although the range is potentially infinite, there are some constraining factors (e.g., the system must be in equilibrium, and the chosen yield criteria must not be violated anywhere). The release of ground stress after excavation during construction will cause the soil to unload. To calculate the deformation and internal force during the construction processes, the ground stress balance calculation is first required to obtain the initial geostress state. And then evaluate the initial pore pressure. Two methods are available to generate the initial stresses, gravity loading or the K0 procedure. The K0 procedure may only be used for horizontally layered geometries with a horizontal ground surface and, if applicable, a horizontal phreatic level. This parameter is defined as the ratio of the horizontal in-situ stress over the vertical in-situ stress. The following formulas are often adopted [28]:

(1) $$K_{0}=1-\sin\;\phi \;\;\text{for}\;\text{granular}\;\text{soils}$$

(2) $${K_{0}=\left(1-\sin\;\phi \right)\left(OCR\right)}^{\sin\;\phi }\;\text{for}\;\text{cohesive}\;\text{soils}$$

where φ, and OCR are respectively the internal friction angle and the over-consolidation ratio of the soil. The overconsolidation ratio (OCR) is defined as the ratio of the maximum past pressure to the existing effective overburden pressure. In general, for NC (Normally Consolidated) clays, OCR = 1 and for OC (Over Consolidated) clays, OCR > 1. K0 is greatly influenced by geological factors such as the soil type, the groundwater configuration [29]. In this paper, the complex shape of the soils layers will induce the use of gravity loading to generate the initial stress state. The pore pressure (Table 2) in all soil layers are evaluated by field measurements. However, this method doesn't take into consideration the working load induce by the machinery during construction time [30]. used a two-stage method to study the behavior of pile group with rigid elevated. Significant results were observed on the behavior of tunneling induced pile response [31]. took into account the coupling effect of longitudinal and lateral deformation and established the governing differential equations for both single piles and pile groups. The working loads, acting on pile heads into account, were taken into account and overcame some deficiencies of existing methods.

**Fig. 9:**
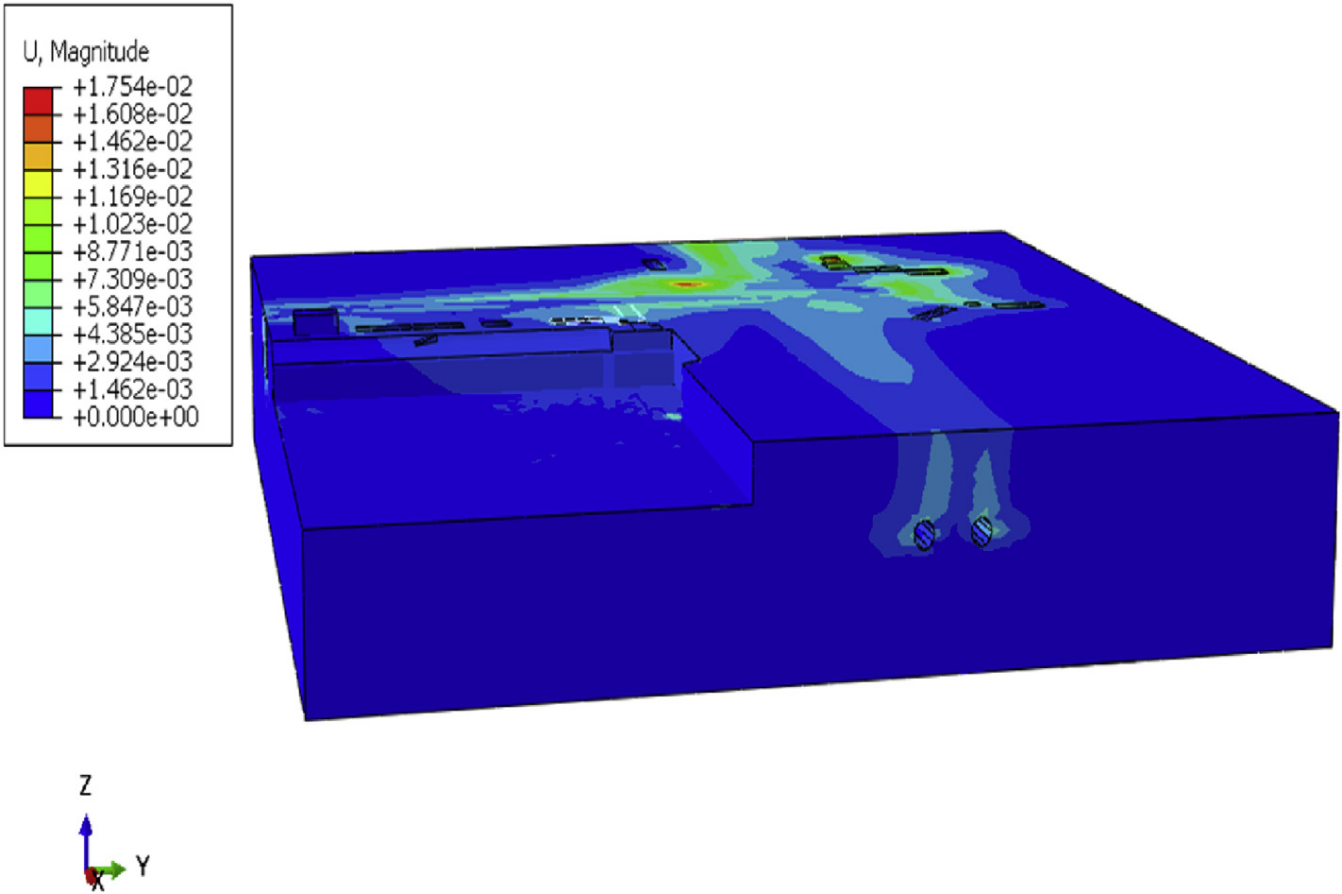
Site construction initial state deformation.

The initial displacement ideal condition is zero. Due to the accuracy and complexity of the model, a small initial value should be calculated, and the initial value used for error correction (Fig. 9). Initial stress conditions, including gravitational loading, may also be given, and a water table may be defined for effective stress calculations.

**Fig. 10:**
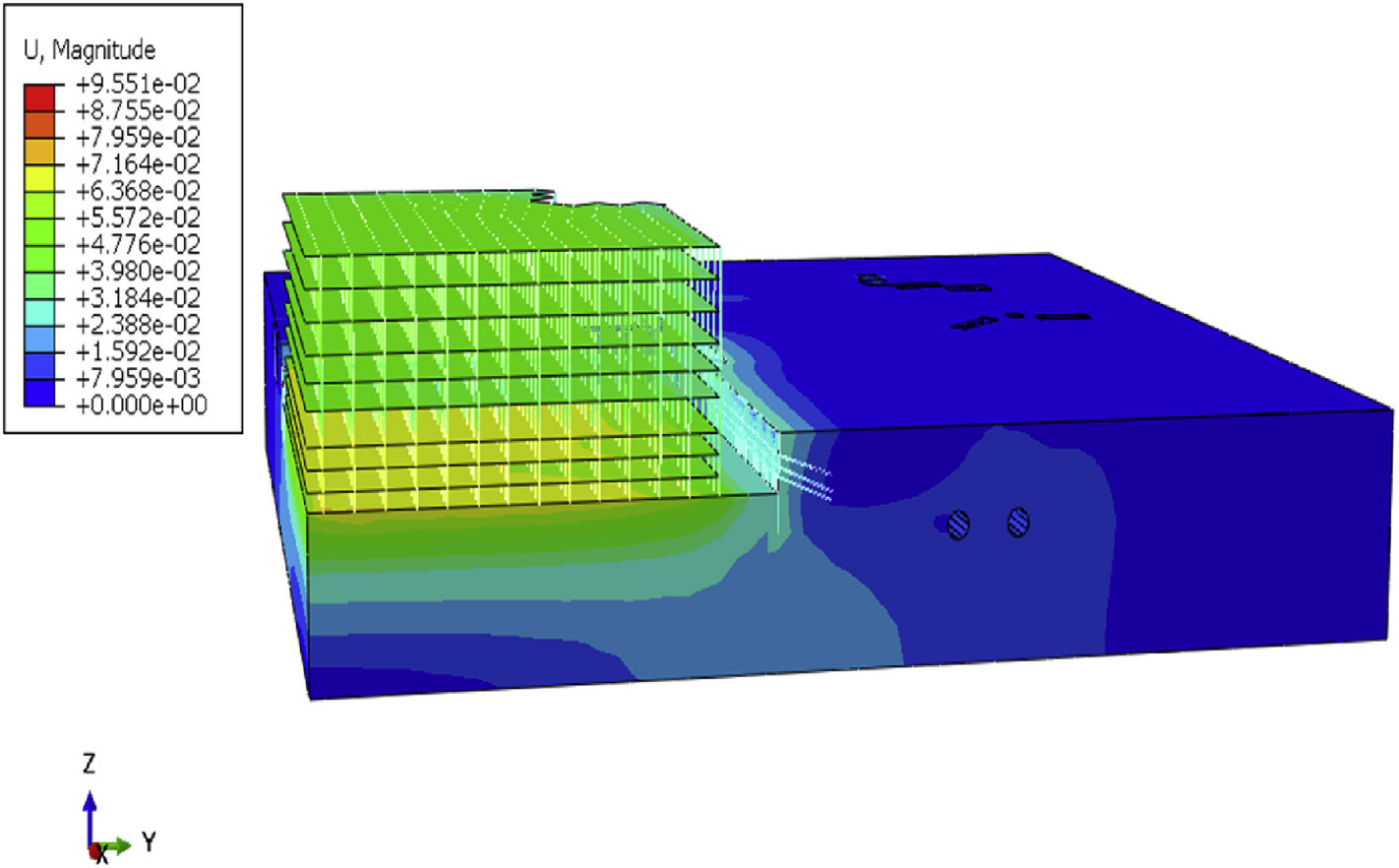
Defomation of the overall model after completion of the project.

After the definition of the initial site condition, begin the stage calculation to simulate the foundation pit repair, communication channel, and building construction. Displacement and stress fluctuation will be observed in the model due to loading conditions variation from step to step calculation. After completion of the building, the maximum displacement on the overall model is 95.51mm (Fig. 10).

### 4.2 Evaluation of monitoring point displacement and reliability analysis

During the construction sequence, some points at the top of the sheet piles walls were chosen, and their deformation evaluated on site using a total station. These values were compared to the numerical method ones to verify the reliability of the results. The displacement values were evaluated after completion of the foundation pit (before the actual construction). In Table 3, the numerical analysis results almost approached the actual measured values. The walls have been modeled as piles in this simulation. This assumption will induce error related to the nature of the walls because it does not provide its real behavior. Also, some damages occurred in the walls to induce its reparation. This fact will slant the monitoring results evaluation. The maximum error value is 61.20% and the minimum value of 43.34%. In light of these results, we can assume the reliability of the numerical method. Furthermore, the controlling measures were proven effective and can potentially provide a reference for similar future projects.

**Table 3:** Numerical simulation and monitoring results.

Technical Measures	Monitoring Results (mm)	Numerical Simulation (mm)	error measured (%)
P1	12.5	24.71	49.41
P2	15.9	34.02	53.26
P3	16.8	38.94	56.86
P4	14	24.71	43.34
P5	13.2	34.02	61.20
P6	17.1	38.94	56.09

### 4.3 Evaluation of the subway tunnels displacement during stage calculation

**Fig. 11:**
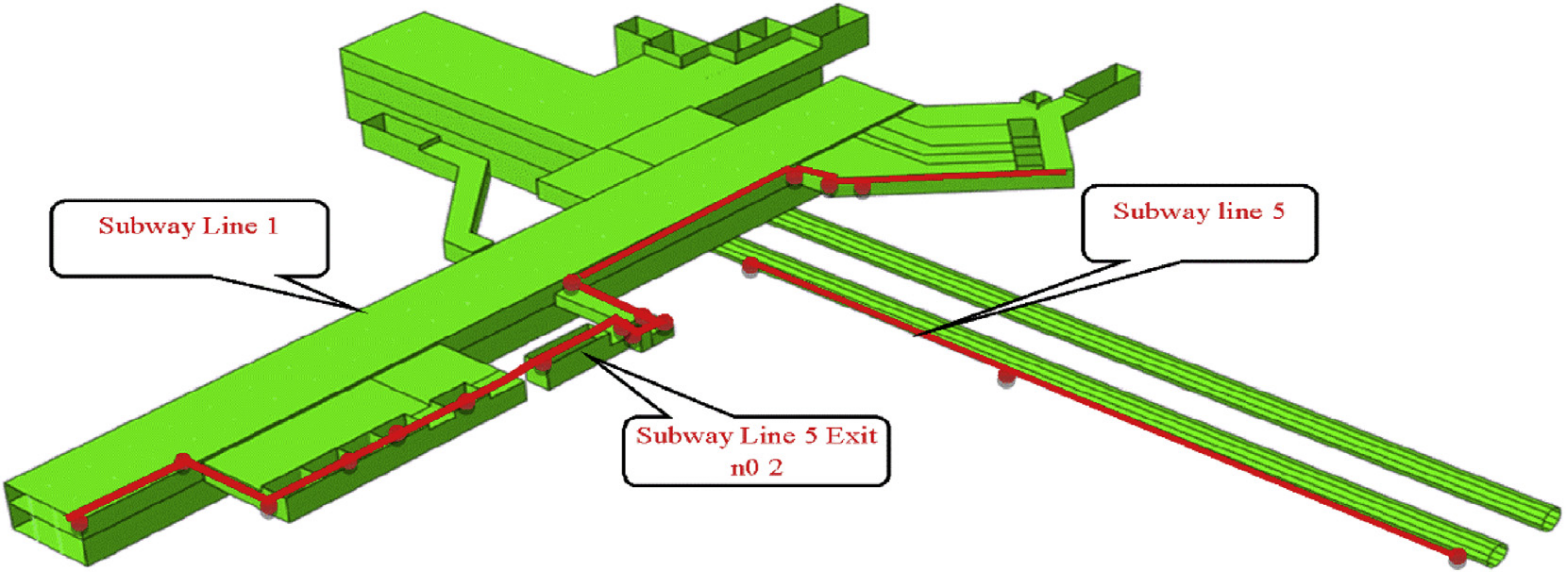
Displacement evaluation range on subways tunnels.

In this section, we will evaluate only the subway lines 1 and 5 displacement behavior in their closest part to the excavation pit. Furthermore, this assumption is reasonable because, during the construction sequence, the largest displacement values are predicted to occur in the subway areas near to the pit foundation. Fig. 11 shows the path of subways line 1 and 5 evaluated in the paper.

#### 4.3.1 Horizontal subway X-displacement

According to the Chinese Code for tunnel safety evaluation under construction sequence, the urban rail transit structure tunnel horizontal displacement warning and control value are less than 10mm and 15mm, respectively. The warning and controlling value of the vertical tunnel displacement are 10mm and 15mm, respectively. In the light of these evaluations measured, it is wise to observe the X-horizontal displacement of the closest part of the subways line 1 and 5 to the construction pit (Fig. 11).

**Fig. 12:**
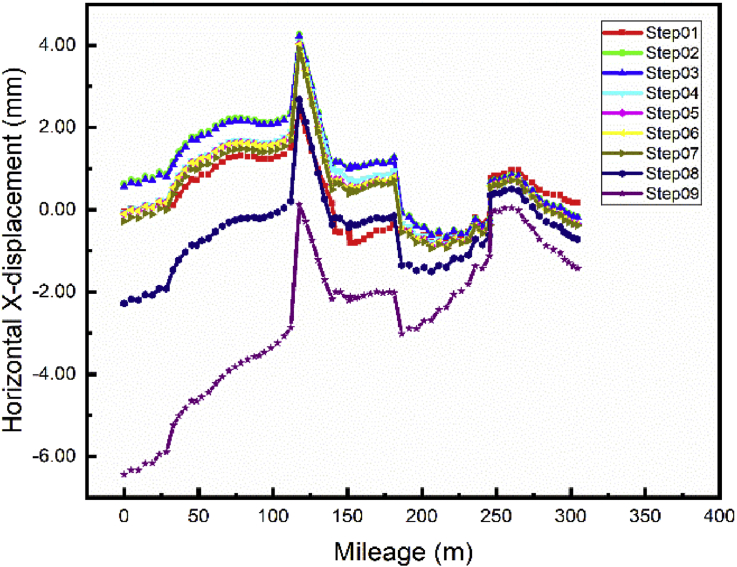
Case 1: Line 1 Path X-lateral displacement.

**Fig. 13:**
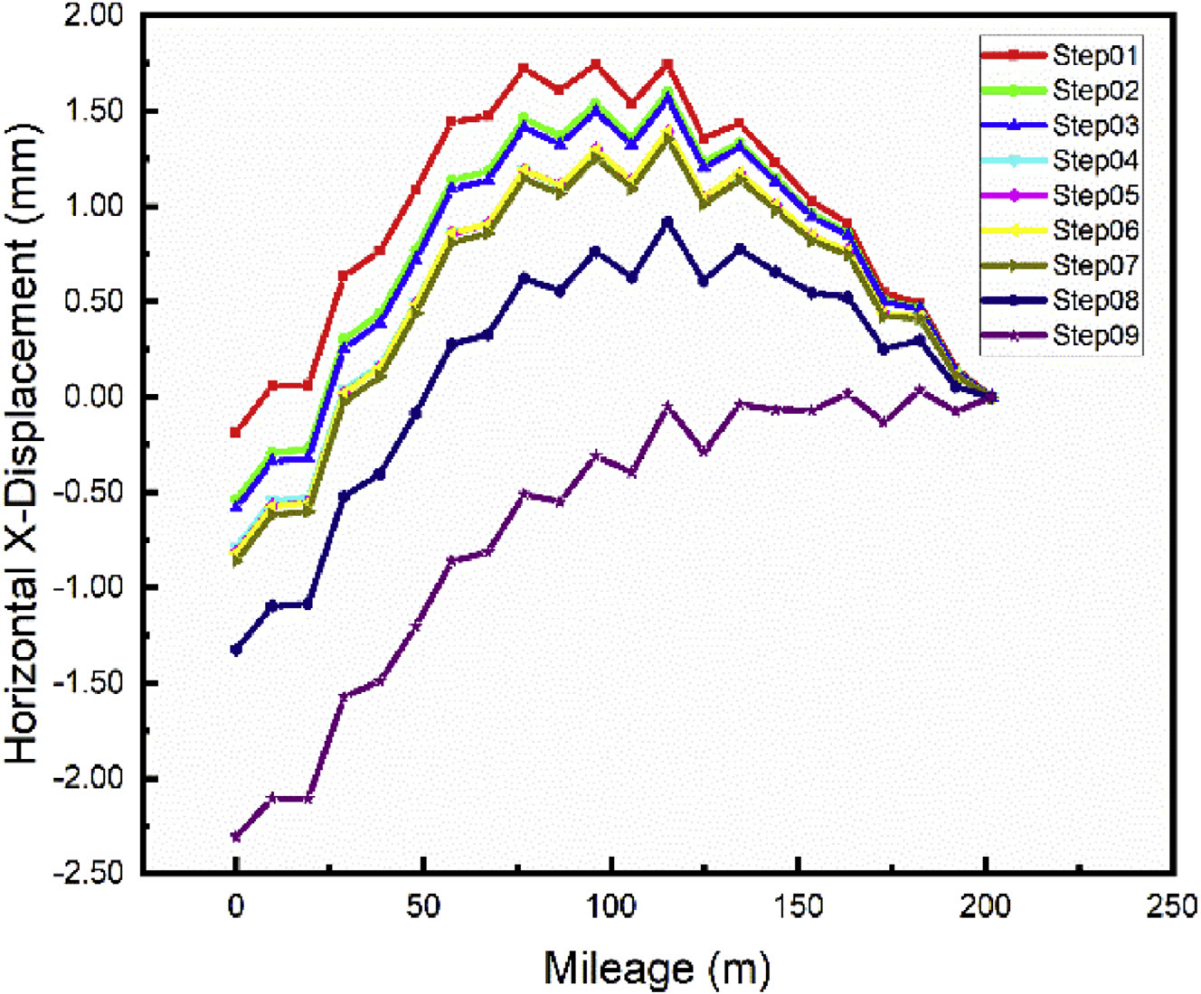
Case 1: Line 5 Path X-longitudinal displacement.

The repair of the main foundation pit has a considerable effect on both subways line 1 and 5 lateral and longitudinal displacement (Figs. 12 and 13). The maximum displacement values were observed in Step02 (Beginning of the main pit repair) 4.264 mm and 1.6mm respectively for subways line 1 and 5. These displacement values decrease to their minimum values during the repair of the foundation pit in step03 (tieback conditions) due to the supporting anchor's cable walls that reduce the displacement of the walls and simultaneously the subways line displacement. After completion of the main foundation pit repair (Step04), begin the construction of the communication channel (Step05 and 06). Fig. 12 and 13 Show that the displacement values stabilizing for all cases during these steps, proving the little effect of the construction of the communication channel on the subways line 1 and 5 respectively lateral and longitudinal displacement. The building construction began in step07 and terminated in step09 after the completion of the communication channel. The construction of the ground floor beams and columns induce little displacement variation the maximum subways line displacement will appear after the completion of the building (Step09) -6.44 mm and -2.31 mm respectively for subways line 1 and 5 in case 1.

#### 4.3.2 Horizontal subway Y-displacement

**Fig. 14:**
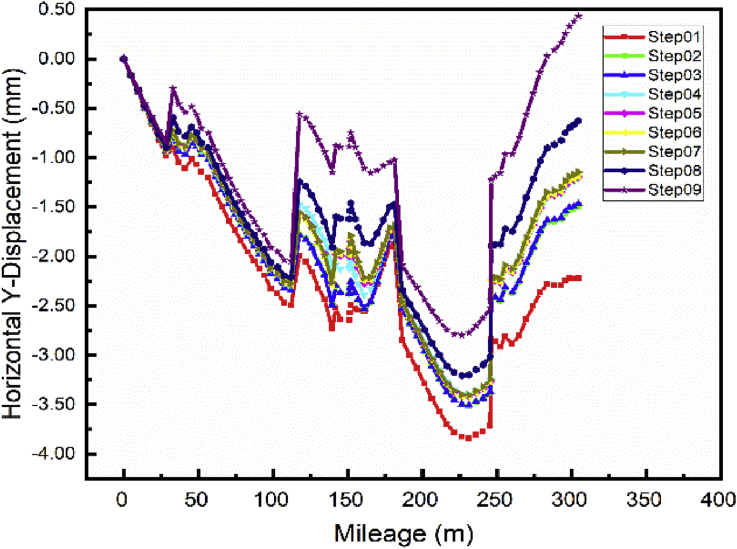
Case 1: Line 1 Path Y-longitudinal displacement.

**Fig. 15:**
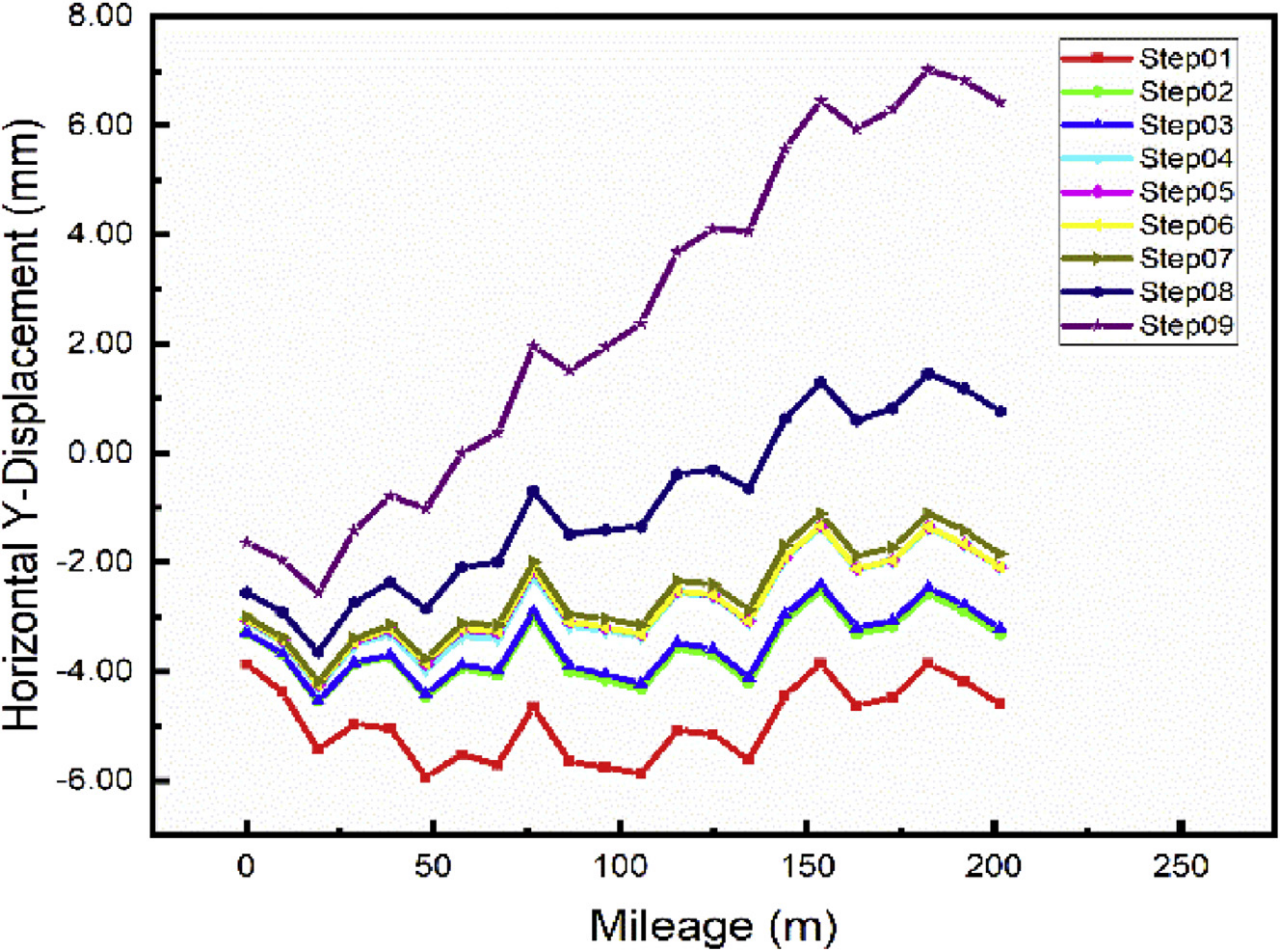
Case 1: Line 5 Path Y-lateral displacement.

The repair of the foundation pit (step02-step04) has little effect on the longitudinal displacement of the subways line 1. During these steps, the maximum displacement variation from step01 to step04 is evaluated at 1.0080mm (Fig. 14). However, the main foundation pit repair has a considerable effect on the lateral displacement of the subway line 5. After completion of these steps, the maximum displacement difference value is estimated at 2.520 mm between Step04 and Step01 (Fig. 15).

After completion of the foundation pit repair (Step04) begin the construction of the communication channel (Step05 and 06). The effect on the longitudinal subway line 1 is minimal (maximum difference value from Step04 to Step06 evaluated at 0.243 mm, respectively, for both cases). The effect is also little on the lateral displacement of the subway line 5 (maximum displacement variation from step04 to step06 evaluated at 0.15 mm). The building construction started in step07 and terminated in step09 after the communication channel completion. This building construction also in e variation on the longitudinal subway line 1 displacement. The maximum displacement variation is observed after completion of the whole building (Step09). That fluctuation value is evaluated at 1.627 mm. However, the building construction will induce higher lateral displacement on subway line 5 (Fig. 15). After the building construction completed, the maximum displacement variation from Step06 to Step09 will be 8.5 mm.

#### 4.3.3 Vertical subway displacement

**Fig. 16:**
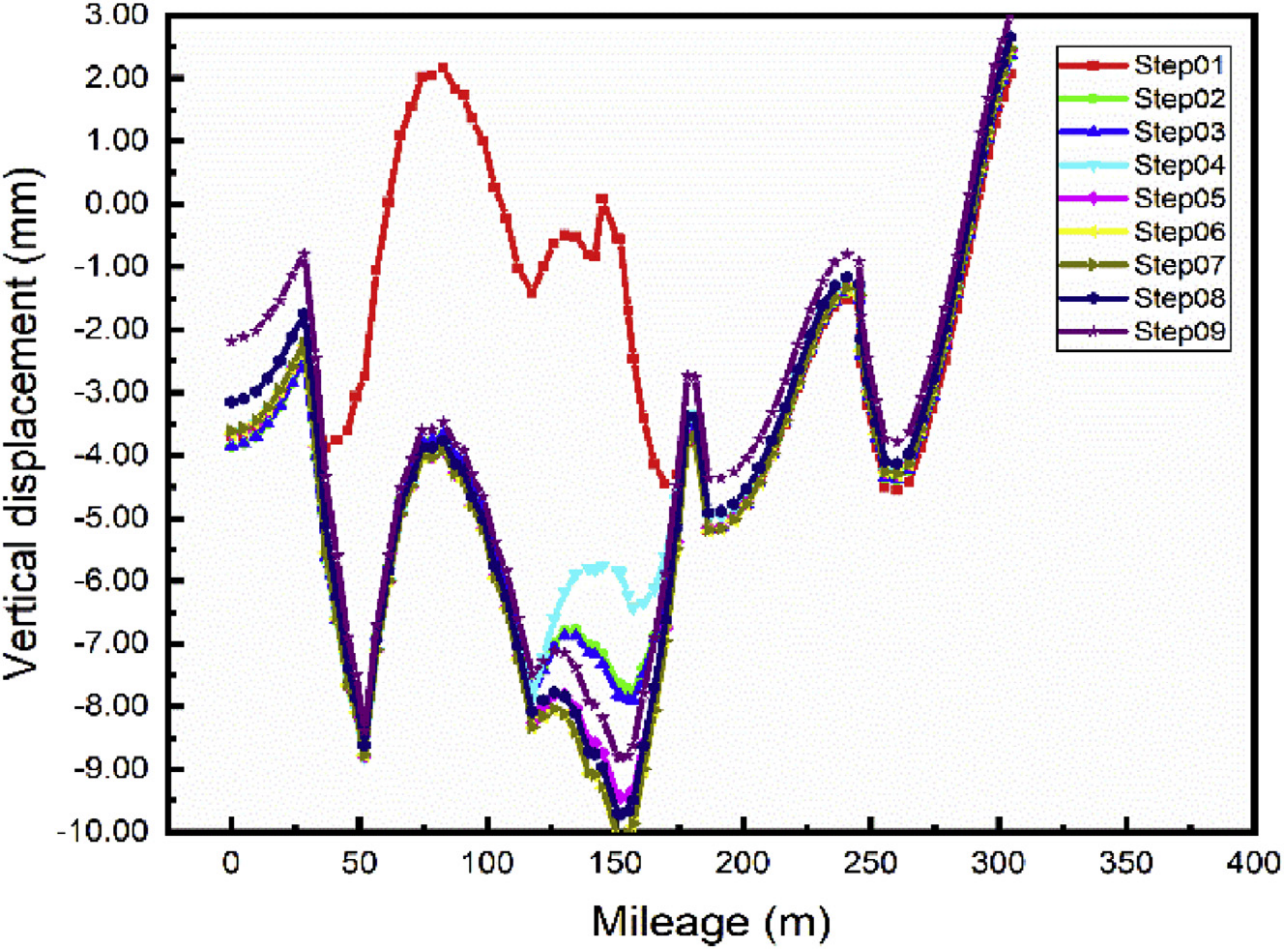
Case 1: Line 1 Path vertical displacement.

**Fig. 17:**
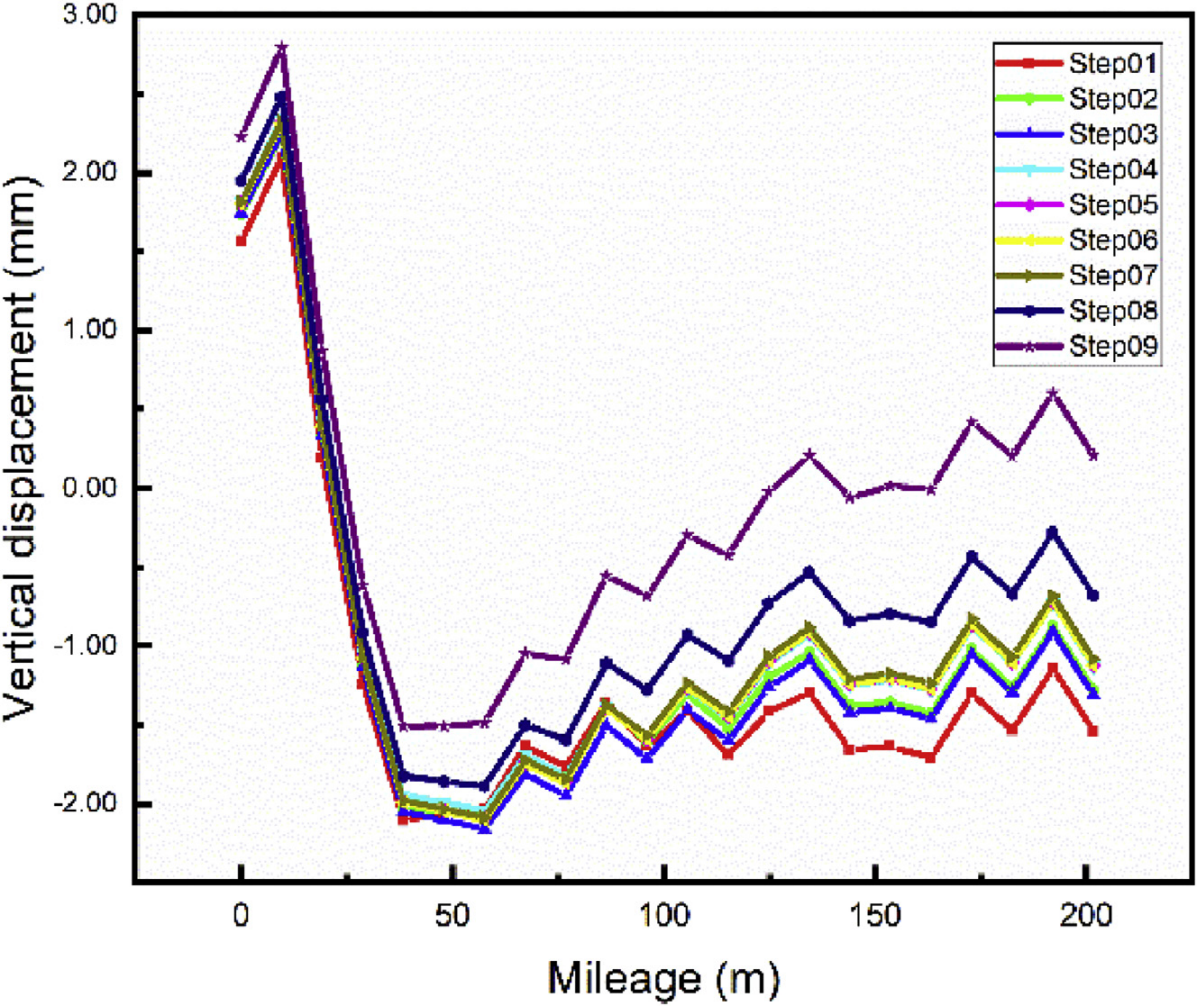
Case 1: Line 5 Path vertical displacement.

The foundation pit repair has a significant effect on the vertical displacement of the subway line 1 (Fig. 16) maximum settlement variation from Step01 to Step04 (Completion of the main foundation pit repair) 6.45 mm. However, these steps have little impact on the settlement of subway line 5 (Fig. 17). Maximum settlement variation from Step01 to Step04 evaluated to 0.43 mm.

After completion of the foundation pit repair (Step04) begin the communication channel construction (Step05 and 06). The end of these sequences results with a maximum displacement variation from Step04 to Step06 to 4.25 mm inducing a maximum subway line 1 displacement evaluated at -10.12 mm. In contrast, these sequences cause little modification on the vertical displacement of subway line 5.

The building construction began in step07 and terminated in step09 after the completion of the communication channel. Completion of the building induces little subways to line 1 and 5 settlement variation from Step06 to Step09. Maximum variation during these phases 1.49 mm and 1.33mm respectively.

## 5 Study area

### 5.1 Cases study

In this paper, two different communication channel construction procedure was investigated. The construction procedure variation was observed from step04 to step06 (start and end of the communications channels construction). This part will focus on the maximum displacement in the path chosen (Fig. 11). This communication section links the mall (building structure) and, the subway line 1 exit n0 2. Study the effects of these two different construction procedure on the subway line 1 exit structure can give a surer and reasonable conclusion in the overall displacement of the subway.

**Fig. 18:**
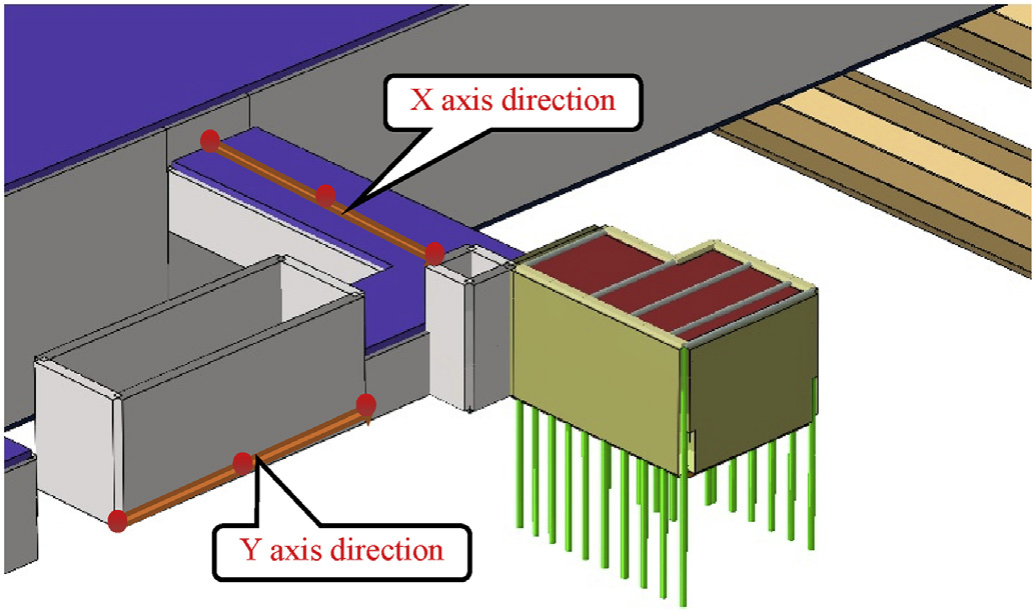
Subway line 1 exit n0 2 and connection channel structure in case 1.

**Fig. 19:**
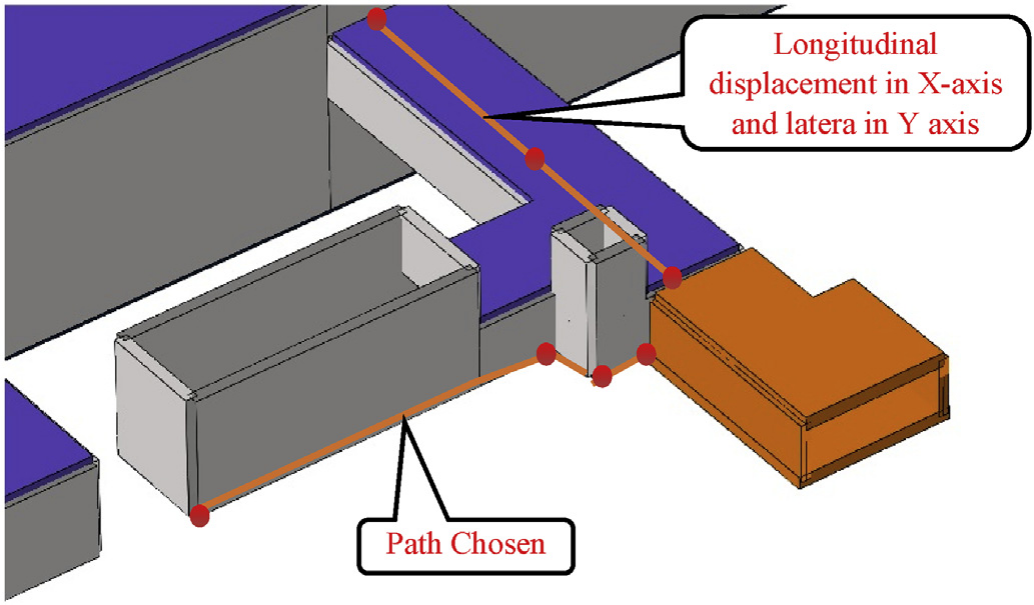
Subway line 1 exit n0 2 and connection channel structure in case 2.

The path chosen induces two kinds of displacement consideration in the horizontal axis (X and Y) and can be considered as lateral or longitudinal relatively the situation (Figs. 18 and 19).

**Fig. 20:**
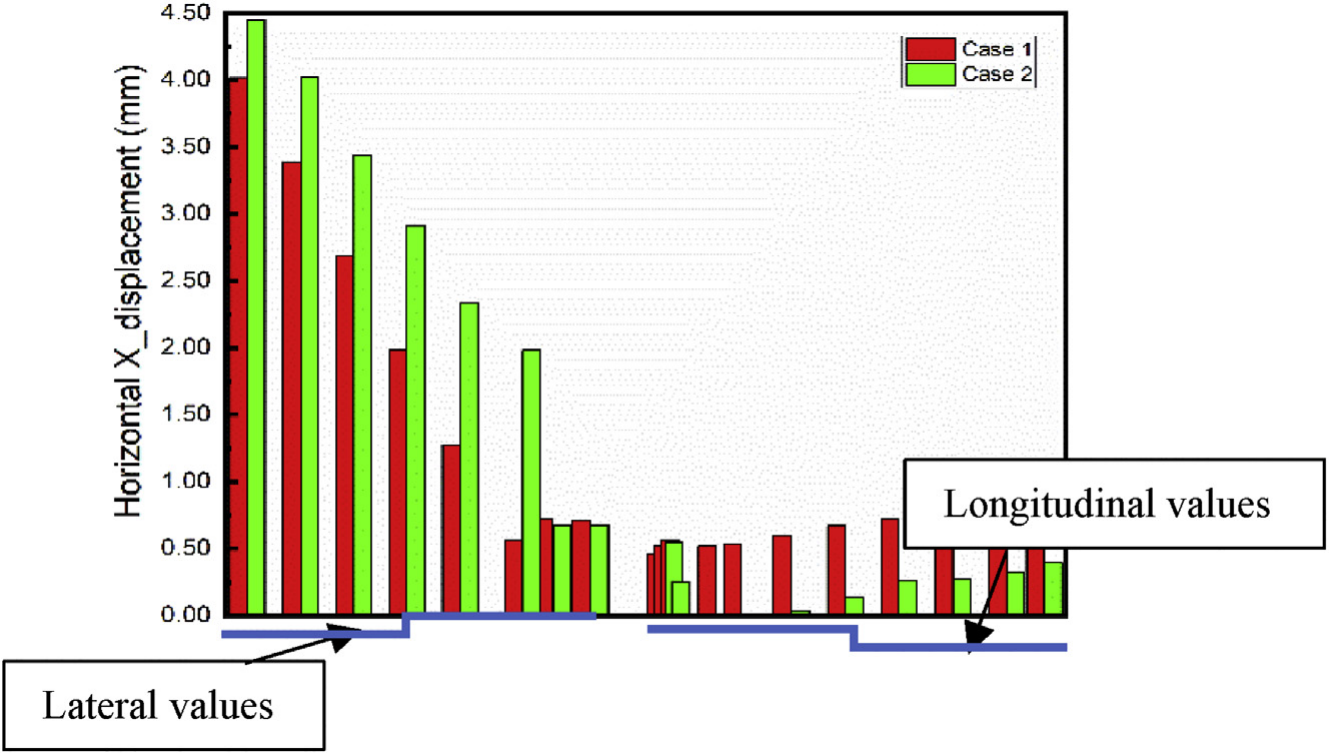
Horizontal X displacement in exit structure path chosen.

**Fig. 21:**
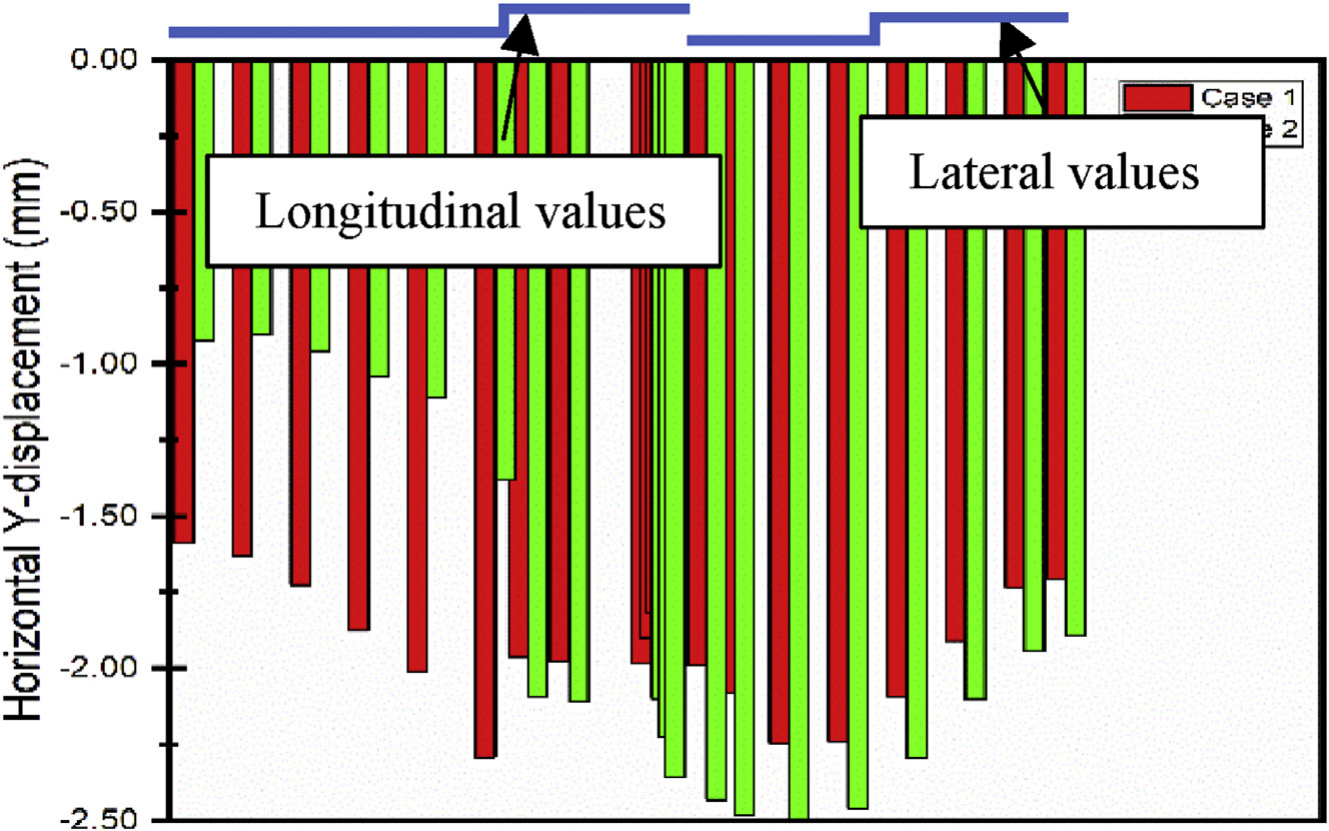
Horizontal X displacement in exit structure path chosen.

**Fig. 22:**
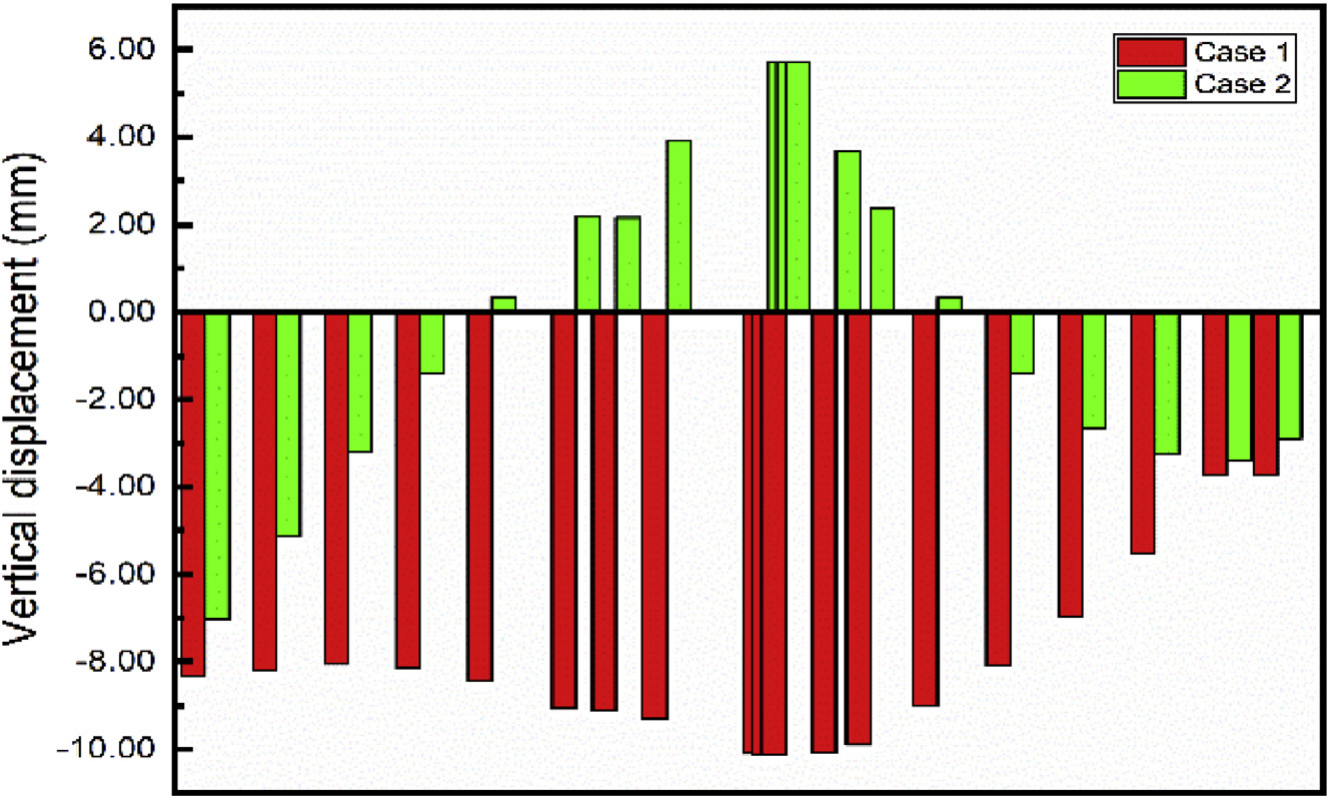
Horizontal X displacement in exit structure path chosen.

As mentioned in the previous part, the communication channel structure construction process started in step04 and ended in step06. Conclusions about the two methods chosen could be reasonably drawn by looking at the displacement on the exit structure after the end of the Step06. Figs. 20 and 21 show the displacement behavior in both cases after completion of the communication channel. The lateral displacement of the subway line exit structure is smaller while using retaining wall and strut to support the soil after excavation than doing the sloped excavation. That case induces mimimum volume soil remove to proceed to the construction than doing sloped excavation. This volume soil to remove diminution induces the reduction of the pit size, which will result in the diminution of the displacement of the soil and surrounding structures near the pit [7]. Installing retaining walls with struts to increase the stability of the walls reduces the horizontal displacement of the soil surrounding the excavation pit. This surrounding soil movement induces the tunnel structure movement, so if the horizontal soil movement is reduced, it will condition the subway tunnel displacement reduction. Similar results ware obtain by Seo [32]. As the bottom supports (struts or anchors) were removed to build the underground structure, additional surface settlement and inward bulging of the wall occurred. However, vertical tunnel displacement is more important in case 1 than case 2 (Fig. 22). In case 1, Installing the retaining walls reduce the horizontal soil displacement this will generate vertical forces due to the pressure induced by the contact between the walls and the soil. These verticals forces will make additional vertical displacement of the surrounding soil near to the walls.

### 5.2 Parametric study

After the cases study, we focus on the case 1 to observe the behavior of the exit structure path displacement chosen while varying the communication channel wall thickness, strut and, pile cross sections.

**Fig. 23:**
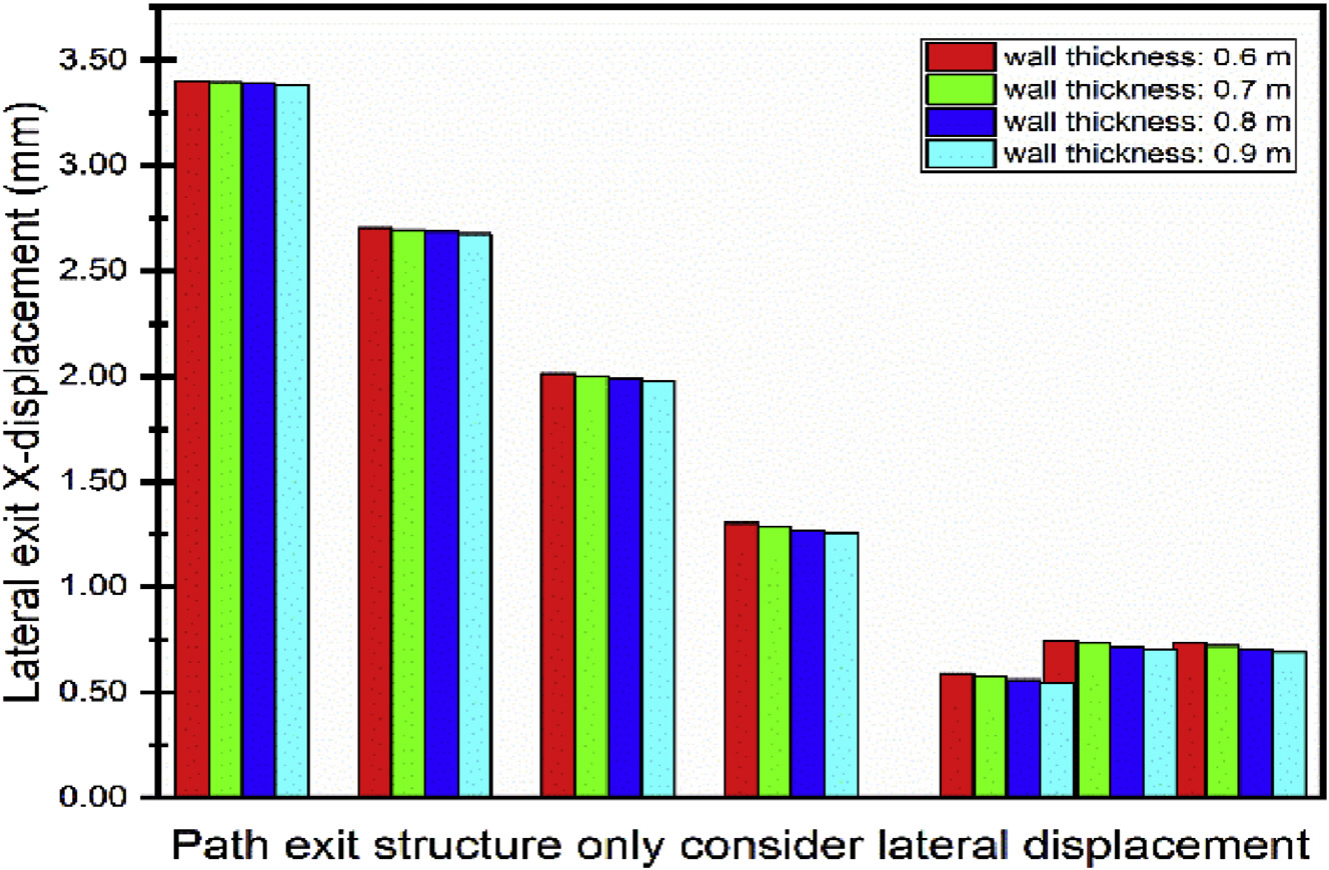
Displacement variation in exit structure path chosen.

The first part of this section will focus on different walls thicknesses effects on the displacement of the exit structure. Fig. 23 shows the lateral outward (direction toward the excavation pit) exit structure decreases due to the augmentation of the thickness of the walls. Similar results were presented by Hashash [33]. Increasing system stiffness and embedment of the wall into a stiff layer are essential factors in limiting the deformations. However, the lateral inward (opposite direction toward the excavation pit) increase due to the augmentation of the wall thickness.

**Fig. 24:**
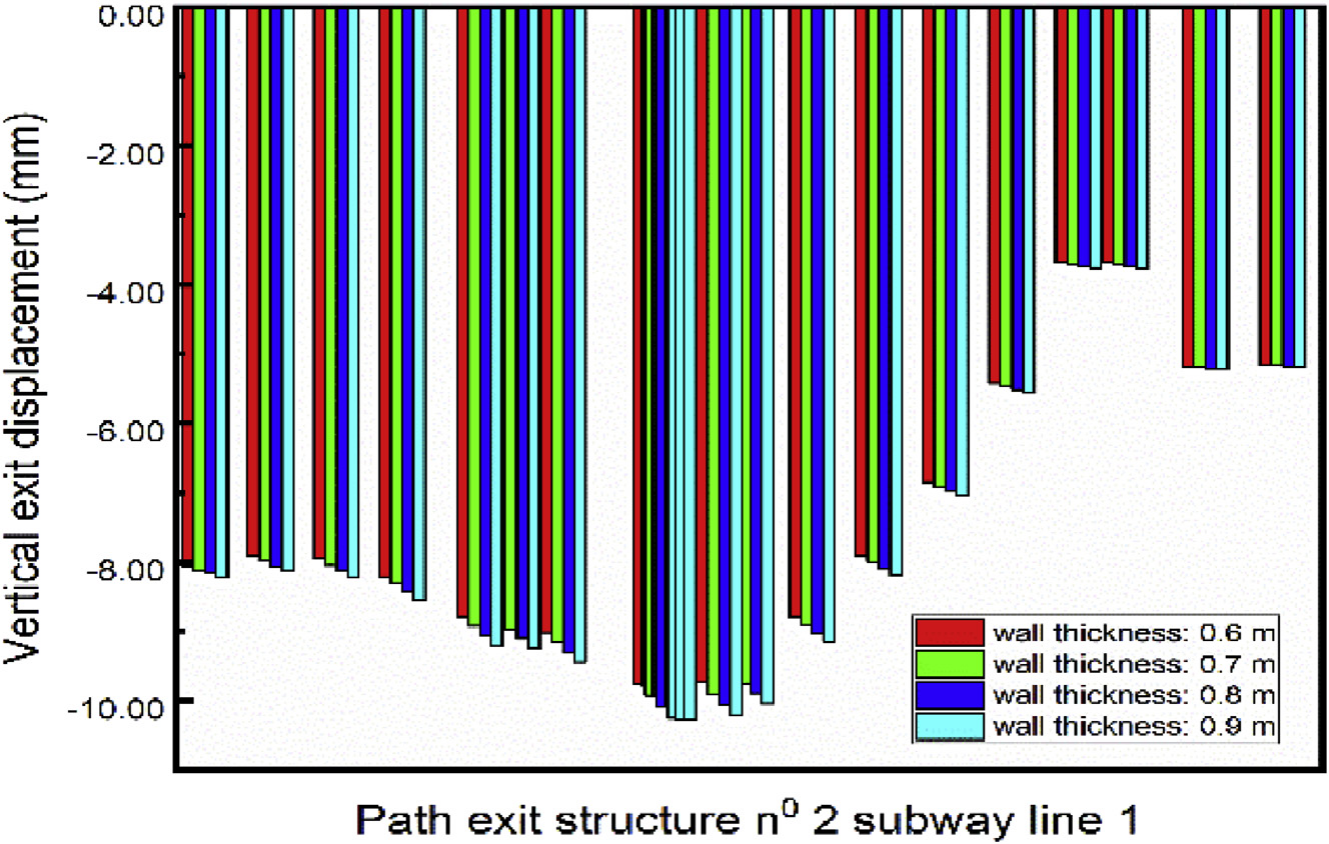
Displacement variation in exit structure path chosen.

The vertical displacement of the exit structure increase with the thickness of the walls increasing (Fig. 24). Aforementioned is due to the augmentation of the wall weight that intends to move downwards the soil around it, by the way, the subway tunnel.

**Fig. 25:**
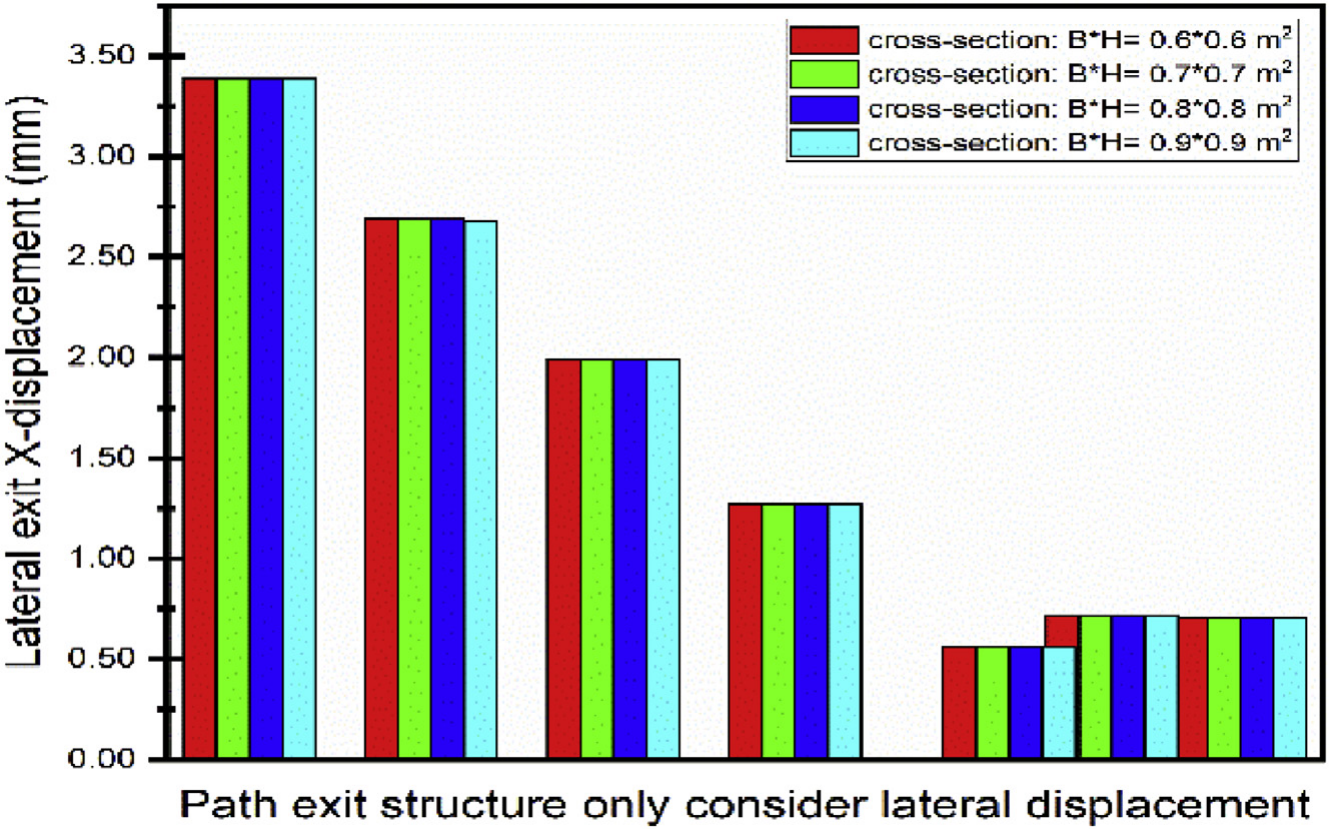
Displacement variation in exit structure path chosen.

**Fig. 26:**
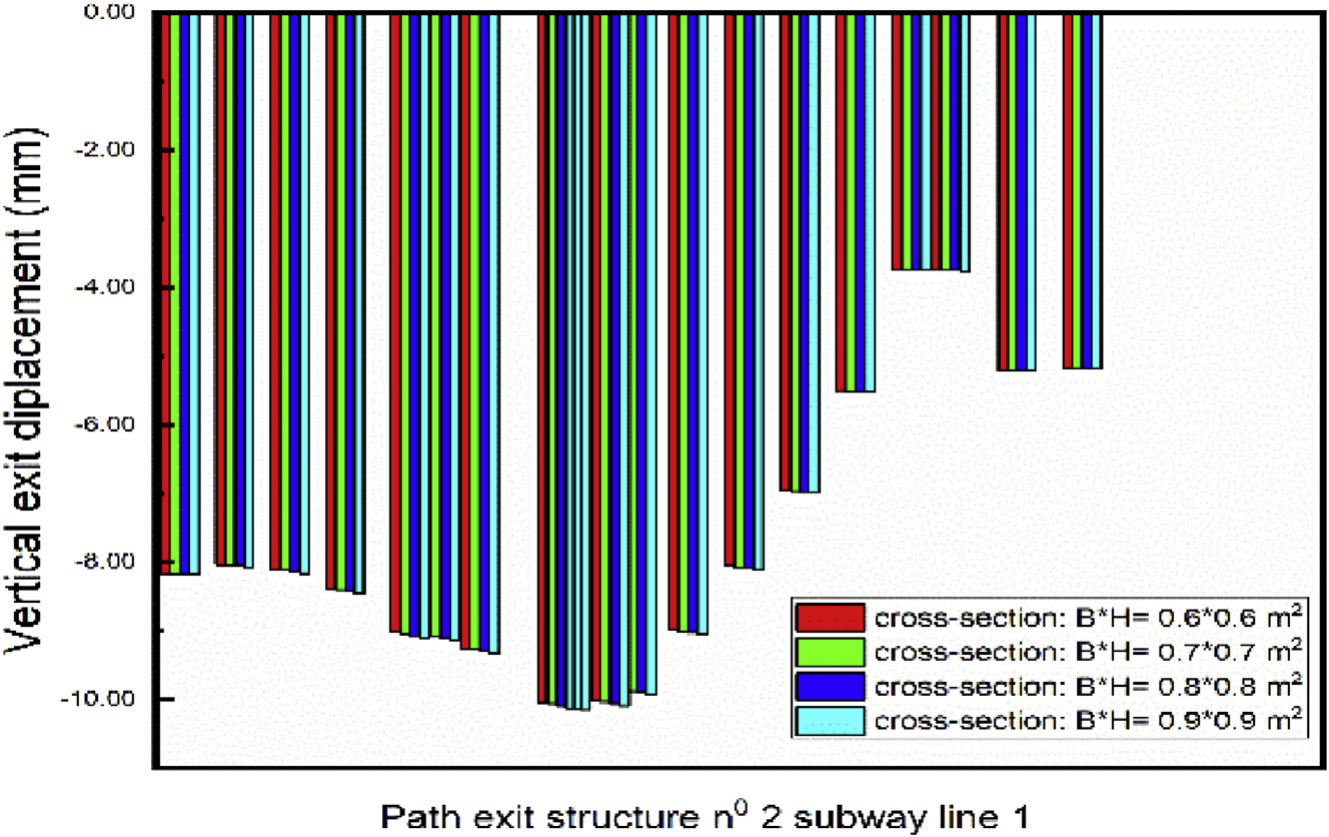
Displacement variation in exit structure path chosen.

The struts had been model like beam element. The shape is a rectangle (width*height). This part focus on the exit structure displacement behavior looking at differents struts cross sections. The lateral displacement variation is not remarkable in both (Fig. 25). This show that the walls thickness variation as much necessary rules in the variation of the exit structure lateral structure than the struts. Fig. 26 shows the exit structure vertical displacement increase with the struts crosses section increases. However, the variation evolution is less important compate to the one obtained from the walls thickness variation.

**Fig. 27:**
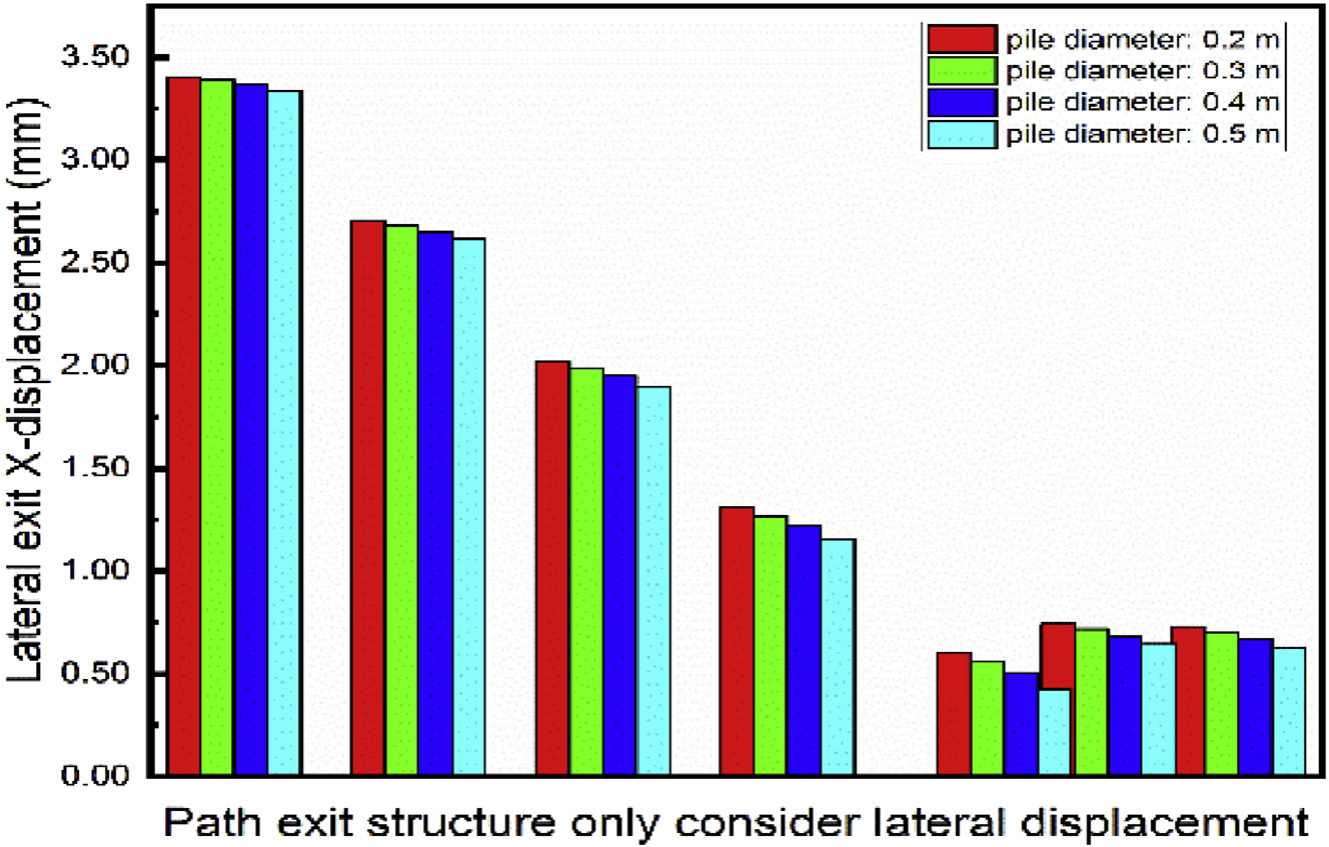
Displacement variation in exit structure path chosen.

**Fig. 28:**
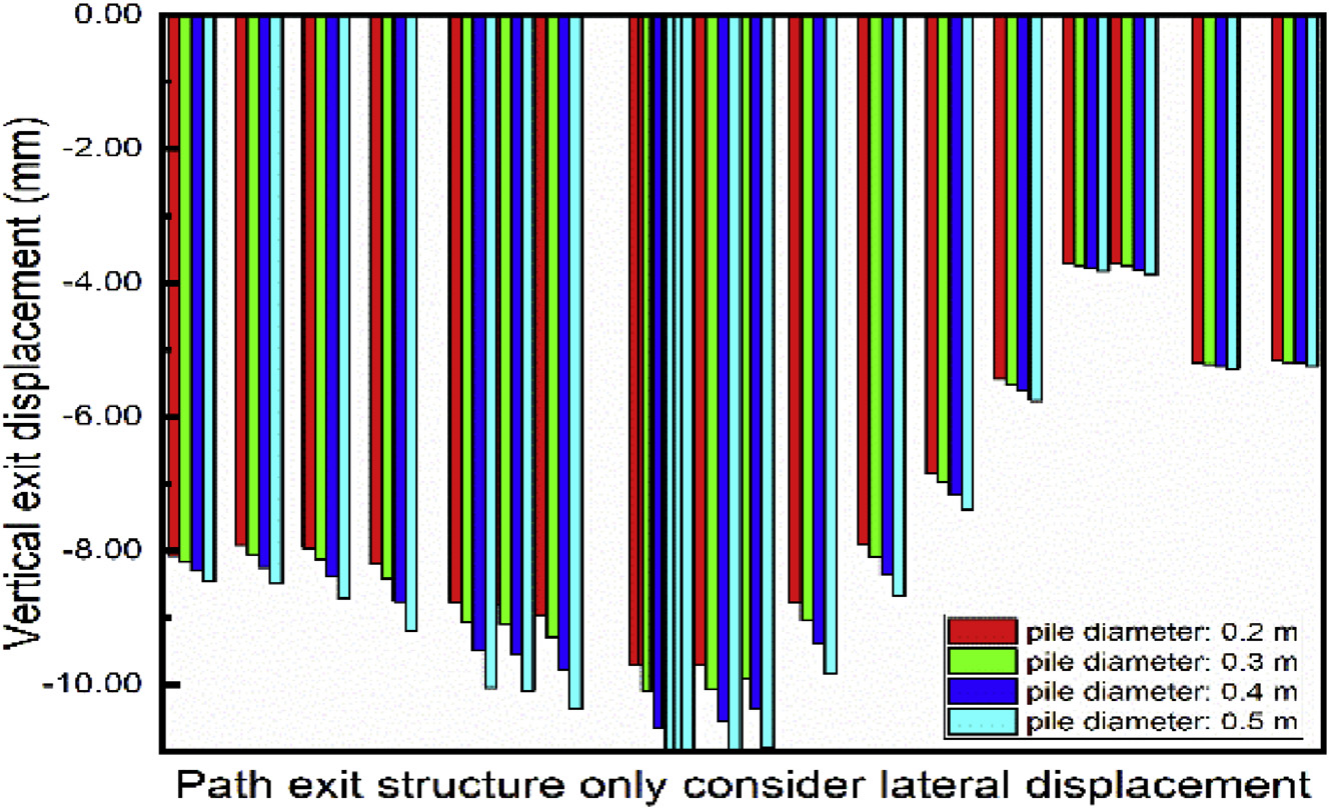
Displacement variation in exit structure path chosen.

The piles cross section had been model as a circle. This section studies the exit structure displacement looking at different piles diameter. Fig. 27. Shows the lateral outward exit structure decrease with the pile's diameter increase. However, unpredictable behavior is observed on the lateral outward exit structure displacement. Significant effects variations in noticed on the vertical exit structure displacement (Fig. 28). The vertical displacement increase with the augmentation of the pile's diameter.

## 6 Conclusions

A numerical simulation on building construction sequence effect on existing subways line was carried out. The conclusions below can be drawn:

1. Installing retaining walls, struts (to increase the stability of the walls) and piles foundation can induce minimum lateral subway line displacement than doing the sloped excavation. In that case, the lateral outward subway line displacement decreases with the walls thickness increases. However, the lateral inward displacement increases with the walls thickness increases. The struts cross-section variation also induce subway line displacement variation. However, the lateral displacement effect of the walls thickness variation is much superior to the one provides by the struts cross-section variation. Although the pile's diameter variation induces variation on the subway structure vertical displacement, this variation is more important than the one noticed in the walls thickness variation. The construction of the walls works more on the reduction of the subway line lateral displacement. However, the pile's construction works more on the subway line vertical displacement. The struts are for the stability of the walls. However, adopting the sloped excavation procedure can induce minimum vertical subway line displacement than the case of installing retaining walls.
2. The difference between the numerical results and the monitoring values could be in an acceptable range to accept the simulation result. A minimum error measured of 43.34% from the numerical simulation and the monitoring results were obtained.
3. Simulate the retaining walls as Sheet piles can induce acceptable results to compare to the field monitoring.

## Declarations

### Author contribution statement

Massamba Fall: Conceived and designed the experiments; Performed the experiments; Analyzed and interpreted the data; Wrote the paper.

Zhengguo Gao: Conceived and designed the experiments.

Becaye Cissokho Ndiaye: Performed the experiments.

### Funding statement

This work was supported by Key Program of National Natural Science Foundation of China (11472029), (11872092) and Beihang university.

### Competing interest statement

The authors declare no conflict of interest.

### Additional information

No additional information is available for this paper.
